# Design, Synthesis, and Cytotoxicity Evaluation of Novel Indolin-2-One Based Molecules on Hepatocellular Carcinoma HepG2 Cells as Protein Kinase Inhibitors

**DOI:** 10.3390/molecules30051105

**Published:** 2025-02-28

**Authors:** Manal M. Kandeel, Mohamed Kamal AbdElhameid, Mohamed Adel, Muhammad Y. Al-Shorbagy, Ahmed T. Negmeldin

**Affiliations:** 1 Department of Pharmaceutical Organic Chemistry, Faculty of Pharmacy, Cairo University, Cairo 11562, Egypt; 2 Department of Pharmaceutical Organic Chemistry, Faculty of Pharmacy, Egyptian Russian University, Cairo 11829, Egypt; 3 Department of Pharmaceutical Sciences, College of Pharmacy, Gulf Medical University, Ajman 4184, United Arab Emirates; 4 Pharmacology and Toxicology Department, Faculty of Pharmacy, Cairo University, Cairo 11562, Egypt

**Keywords:** HepG-2 cells, MCF-7, isatin, quinazolines, protein kinases, tyrosine kinase, CDK

## Abstract

A series of indolinone-based derivatives were designed and synthesized using the hybrid pharmacophoric design approach as cytotoxic kinase inhibitors. The cytotoxic effects of the designed molecules were tested against MCF-7 and HepG-2 cell lines. Compounds **9** and **20** were the most cytotoxic, with IC_50_ values against HepG-2 and MCF-7 cells ranging from 2.53 to 7.54 µM. Additionally, compounds **9** and **20** were also found to be slightly more cytotoxic than indirubin with 2.2–2.7-fold higher cytotoxicity with HepG-2 cells. CDK-2 and CDK-4 kinase enzyme inhibition assay showed that compound **9** had a higher inhibitory effect (4.8-fold) than indirubin against CDK-2 and comparable inhibition against CDK-4. Moreover, compound **20** displayed nanomolar inhibitory action against both EGFR kinase and VFGFR-2 enzyme, which were around 8.8- and 5.4-fold higher than the IC_50_ values of indirubin. Compounds **9** and **20** induced cell cycle arrest at the G_1_ phase on HepG2 cells. The levels of the key apoptotic proteins assessed revealed elevated levels of the Bax/Bcl-2 ratio, which in turn initiated the caspase3/7 cascade that led to the activation of both intrinsic and extrinsic apoptotic pathways. The cell cycle inhibitory proteins p53 and p21 were significantly upregulated upon treatment with compounds **9** and **20**. The docking results revealed that compound **9** exhibits stronger binding affinity to CDK-2 than indirubin, and compound **20** showed a similar binding mode to sorafenib with VEGFR-2.

## 1. Introduction

**Figure 1:**
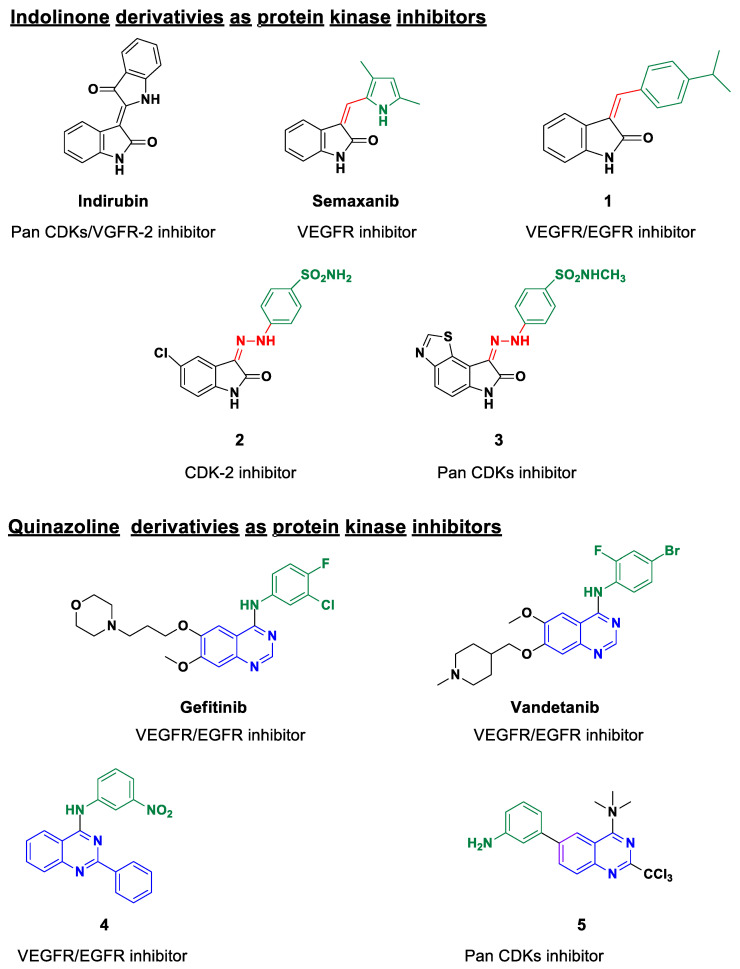
Chemical structures of several lead protein kinase inhibitors containing C3-substituted 2-indolinone or quinazoline ring systems. The indoline ring is shown in black, the bridge group in red, the aryl groups in green, and the substituted quinoline rings in blue.

Hepatocellular carcinoma (HCC) is considered among the most aggressive types of cancer and is spread worldwide. The incidence of HCC is expected to increase in the following 20 years by about 60% in both females and males at different ages. Cancer cells including HCC are characterized by overexpression of receptor tyrosine kinases (RTKs) such as epidermal growth factor receptor (EGFR) and vascular endothelial growth factor receptor (VEGFR) that control cell proliferation and angiogenesis in cancer cells [1]. Overexpression of VEGFR and EGFR results in sustained angiogenesis and up-regulation of cyclin-dependent kinases (CDKs), leading to proliferation and cell cycle dysregulation [2]. The resistance to apoptosis in cancer cells in HCC is due to overexpression of both VEGFR and CDK enzymes. Initiation and progression of adenocarcinoma, such as HCC, depends on more than one signaling pathway. Several chemotherapy protocols that include a combination of drugs targeting more than one receptor are more successful in such types of tumors [3]. Indirubin (**Ind**) (Figure 1) is a bis-indole natural compound reported as a multi-target kinase inhibitor with the ability to inhibit several kinases, including EGFR, VEGFR, and CDKs. In a previous study, Indirubin showed the ability to inhibit cell proliferation, migration, invasion, and angiogenesis of HCC HepG2 tumor-derived endothelial cells, suggesting the ability of indirubin to treat HCC [4]. Indirubin structurally has an indolinone fragment and is considered as a lead molecule for several anticancer drugs, such as Semaxanib (Figure 1) and Sunitinib [5,6,7,8,9]. Semaxanib has been used in the treatment of colorectal cancer, while Sunitinib has received the FDA approval for the treatment of renal and gastrointestinal cancers [10].

Structural analysis of several indirubin analogs has shown that their kinase inhibitory activities rely on the presence of an isatin ring merged with various aryl groups, where the indolinone group acts as the main pharmacophoric moiety [11]. The indolinone ring plays an essential role as a competitive inhibitor of ATP binding to its catalytic pocket of the protein kinase enzymes. According to the ring substitution pattern, site of linking with other aryl groups, and chemotype of merged aryl rings, the protein kinase selectivity of the cytotoxic indolinone inhibitors has been defined [12]. The C3 position of the indolinone ring was found to be the most reliable position for connection with other fragments. Several C3-aryl substituted indolinones (or C3-substituted isatins) have been reported in the literature due to their relative stability compared to C2-substituted indolinones, where they demonstrated cytotoxic activity against different cancer cell lines [13,14,15]. Among the C3-substituted indolinones are C3-arylidene derivatives, where a methylene linker connects the indolinone ring to a C3-aryl substituent, including Semaxanib and Sunitinib. Semaxanib, the pyridinone derivative of Sunitinib, has displayed 3-fold more cytotoxic activity than sunitinib against one of the HCC cell lines, HepG2 cells [16]. Hydrazone has also been utilized as a successful linker between the core indolinones and the aryl ring, which has led to several indolinone-based hydrazones, such as compounds **2** and **3** (Figure 1), with promising cytotoxic activities against different cell lines [17].

Substituted quinazoline rings have been recognized for their cytotoxic activity towards different cell lines, and several of them have been approved for treatment of different types of cancers, while others are at different stages of clinical trials. Multiple variations of substituents on the quinazoline ring, including 4-aminoquinazoline, which acted as a core ring system in several anti-cancer agents, have been reported [18]. Gefitinib and vandetanib and compound **4** (Figure 1) are examples of the 4-aminoquinazolines that have been used for the treatment of metastatic non-small cell lung cancer. Gefitinib has shown a synergistic effect with irinotecan on suppressing hepatocellular carcinoma [19,20]. Compound **5**, bearing a quinazoline moiety, has been reported as a pan-CDK inhibitor with a dual affinity toward RTK and CDK enzymes.

**Figure 2:**
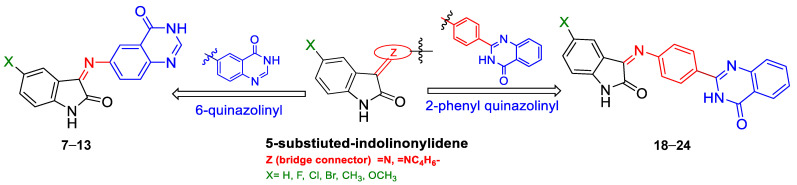
Design of target compounds **7**–**13** with an imino linker and C6-quinazolin-4-one ring and compounds **18**–**24** with the phenyl ring spacer and C2-quinazolin-4-one ring. The indolinone core ring is shown in black, the bridge connecting groups are shown in red, and the quinazoline ring system is shown in blue.

Prompted by the previous findings and based on a hybrid pharmacophoric design approach, several compounds were designed and synthesized in this work with two novel structural models designed by merging different C3-aryl substituted indolinones with several quinazoline fragments. The rationale for the design behind these novel molecules was based on the reported inhibitory activities for both fragments and that combining varying indolinone systems with different quinazoline rings would lead to several novel dual RTK and CDK inhibitors with potentiated cytotoxic effects [21]. The designed compounds carry pharmacophoric C3-indolinone fragments carrying different substituents on the C5 of the indolinone rings and fused with different quinazoline or quinazolinone rings through various linkers capable of forming hydrogen bond(s) (Figure 2). In the first model (compounds **7**–**13**), a short and rigid imino linker was used to connect the indolinone pharmacophore to a C6-quinazolin-4-one ring. The second model (compounds **18**–**24**) has an extended connector with a phenyl ring spacer and an imino group connecting the indolinone core ring system to different C2-quinazolin-4-one ring systems. The newly developed compounds **7**–**13** and **18**–**24** were evaluated for their cytotoxic effects in vitro on HepG-2 and MCF-7 cancer cell lines. Additionally, this study assessed the most cytotoxic compounds, **9** and **20**, against CDK-2, CDK-4, VEGFR-2, and EGFR to determine their IC_50_ values. Furthermore, compounds **9** and **20** were examined for their ability to inhibit cell growth and induce apoptosis through DNA flow cytometry and annexin V analysis on HepG-2 cells. ELISA measurements of cell cycle regulatory proteins p53 and p21 were conducted for compounds **9** and **20** at their IC_50_ values. Simultaneously, the apoptosis indicators (Fas-L, Bax/Bcl-2 ratio, and caspase 3/7) were also evaluated for compounds **9** and **20**. Moreover, a molecular docking study was carried out for the most active molecules at the enzyme binding sites of VEGFR-2 and CDK-2 to explain the observed cytotoxic and inhibitory effects.

## 2. Results and Discussion

### 2.1. Chemical Synthesis of the Target Compounds

**Scheme 1:**
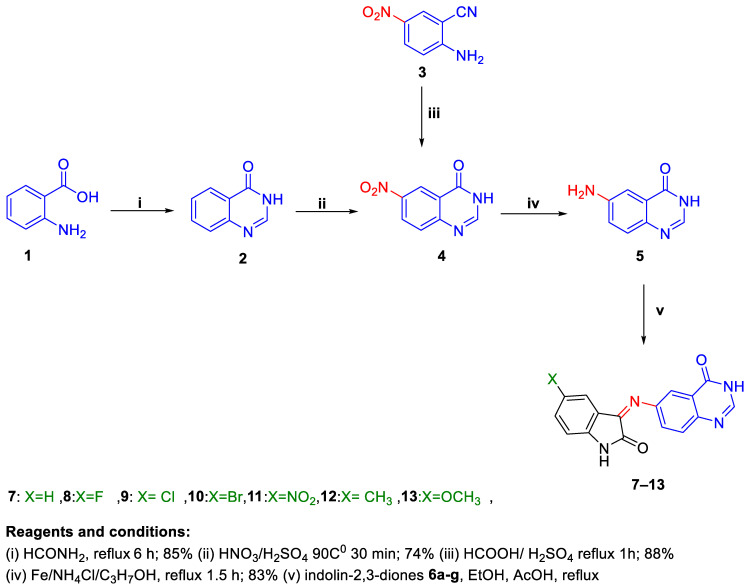
Chemical synthesis of 6-(indolylidonamino)quinazolinone derivatives (**7**–**13**).

**Scheme 2:**
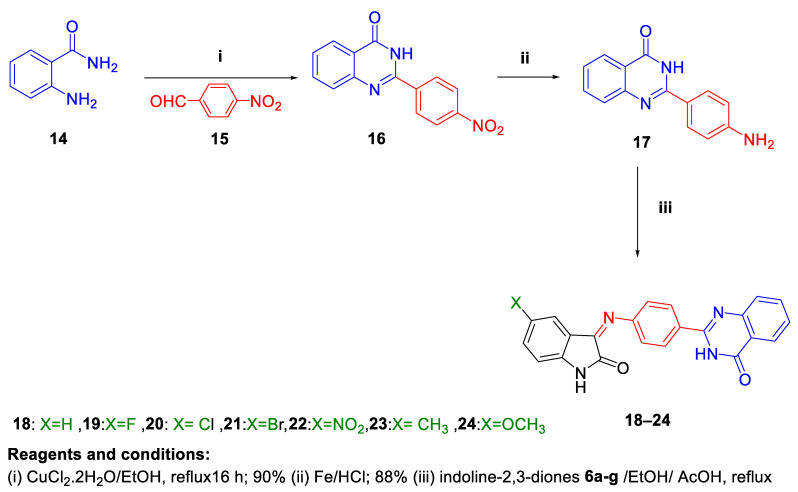
Chemical synthesis of 2-[4-(indolylidoneamino)phenyl]quinazolinones compounds (**18**–**24**).

The synthetic routes employed to synthesize the target derivatives are represented in Scheme 1 and Scheme 2. Anthranilic acid (**1**) was heated with formamide and afforded 4-quinazolinone (**2**) [22], which was nitrated with a mixture of concentrated nitric acid and sulfuric acids to furnish 6-nitroquinazolinone as key intermediate **4** [23,24] (Scheme 1). Intermediate **4** was also obtained through cyclization of 2-amino-5-nitro-benzonitrile (**3**) with formic acid with a better yield than the previous route [22,23,24]. Intermediate **4** was then reduced using a mixture of iron and ammonium chloride in isopropanol, producing 6-aminoquinazolinone (**5**) [25]. Synthesis of the target 6-(indolylidonamino)quinazolinone compounds (**7**–**13**) was achieved via the reaction of the aminoquinazoline (**5**) with various indolinediones (**6a**–**g**) by using ethanol as a solvent in the presence of acetic acid as a catalyst (Scheme 1).

The electronic effect of the group of the isatin ring had a slight impact on the yield of the final products. The highest yield percentage was observed with compound **10** (70% yield), in which 5-bromo-isatin was used, bearing bromine as an electron-withdrawing group. On the other hand, the presence of electron-donating groups, such as in 5-methoxy isatin and 5-methyl isatin, gave the lowest yields in the series with 50% and 48% yields in compounds **12** and **13**, respectively. IR spectra of the observed products showed characteristic absorption bands at the ranges of 3154–3199 cm^−1^ and 3200–3258 cm^−1^, corresponding to NH groups. In addition, bands at the ranges of 1665–1694 cm^−1^ and 1650–1675 cm^−1^ were also observed, indicating the presence of C=O groups. The ^1^H-NMR spectra of compounds **7**–**13** showed two exchangeable singlet signals at *δ* 10.30–11.13 ppm and *δ* 10.75–12.33 ppm, corresponding to the NH protons. Furthermore, ^13^C-NMR spectra showed signals resonating in the range of *δ* 162.8–165.4 ppm as characteristic signals of the carbons of the carbonyl groups in compounds **7**–**13**.

Target compounds **18–24** were synthesized according to the synthetic pathway illustrated in Scheme 2. Synthesis was initiated via heating under reflux a mixture of anthranilamide (**14**) with nitrobenzaldehyde (**15**) and Copper (II) chloride in ethanol, which yielded the 2-(nitro-phenyl)quinazolinone intermediate (**16**) [26]. Compound **16** was reduced with iron and hydrochloric acid, leading to 2-(4-aminophenyl)quinazolinone (**17**) [27]. Finally, compound **17** was condensed with several substituted indolinediones (**6a**–**g**) via refluxing in ethanol, using acetic acid as a catalyst to afford the 6-aminoquinoline 2-[4-(indolylidoneamino) phenyl]quinazolinones **18**–**24** (Scheme 2).

### 2.2. Biological Screening

#### 2.2.1. In Vitro Cancer Cell Growth Inhibition

The cytotoxic activities of the newly synthesized molecules **7**–**13** and **18**–**24** were examined against two human tumor cancer cell lines (HepG-2 and MCF-7) using the MTT assay (Table 1) [28]. Indirubin was used as the positive control. The HepG2 and MCF-7 cell lines were selected to evaluate the biological activity of the target compounds due to their relevance in liver and breast cancer research, respectively. Both cell lines express CDK2, a key regulator of the cell cycle, and VEGFR-2, which is involved in angiogenesis and tumor growth. This makes them suitable models for assessing the efficacy of compounds targeting these proteins [29,30,31,32]. Notably, the newly synthesized compounds’ activities against the two cell lines varied noticeably depending on their substitution patterns.

The results of the cytotoxicity screening showed that compound **9**, which has a chlorine substitution, displayed the highest cytotoxicity among the first series of compounds (**7**–**13**) against the HepG2 (IC_50_ = 2.53 µM) and MCF-7 (IC_50_ = 7.54 µM) cell lines (Table 1). While Compound **13** activity against MCF-7 was less evident (IC_50_ > 100 µM), it did demonstrate noticeable inhibition against HepG2 (IC_50_ = 18.86 µM). Chlorine-substituted compound **20** had the best inhibitory effect among the compounds of the second series (**18**–**24**) on the HepG2 and MCF-7 cell lines with IC_50_ values of 3.08 µM and 5.28 µM, respectively (Table 1). These values were superior to the reference compound (Ind), which displayed IC_50_ values of 6.92 µM and 6.12 µM, highlighting the efficacy of the chlorine-substituted derivatives.

Compounds with electron-donating groups, such as -CH_3_ (compounds **12** and **23**) and -OCH_3_ (compounds **13** and **24**), displayed lower cytotoxicity, with IC_50_ values exceeding 18 µM in most cases. On the other hand, compounds with electron-withdrawing groups, like -NO_2_ (compounds **11** and **22**), showed moderate activity. Notably, compounds with bulky substituents, like -Br (compounds **10** and **21**), exhibited variable activity, with compound **10** showing low activity against MCF-7 (>100 µM) but moderate potency against HepG2 (28.11 µM).

The halogen substituents (-Cl and -F) consistently enhanced cytotoxicity, which could be due to their electronic and steric effects, which facilitated interactions with biological targets. Chlorinated derivatives, particularly compounds **9** and **20**, demonstrated superior activity, suggesting the importance of chlorine as an effective substituent for this scaffold. In contrast, bulky substituents or strong electron-donating groups may introduce steric hindrance or unfavorable electronic effects, reducing activity.

**Figure 3:**
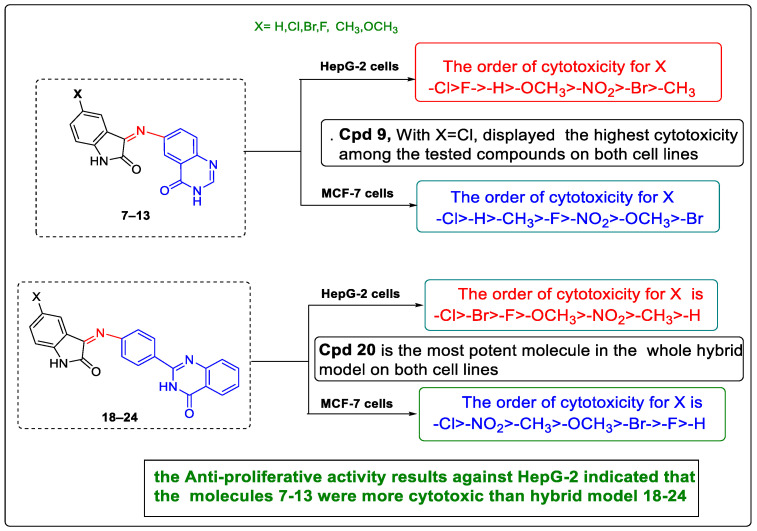
Structural activity relationship (SAR) analysis of synthesized molecules **7**–**13** and **18**–**24** according to the observed IC_50_ values (µM) on HepG-2 and MCF-7 cell lines.

Regarding the influence of structural variations, such as fragment chemotypes, type of connecting bridge, and position of site of attachment at the quinazoline ring, on the cytotoxicity, 5-chloro-isatin was found to be crucial for cytotoxicity since the chloro-isatin moiety was present in the most active compounds (**9** and **20**) (Table 1 and Figure 3). The effects of quinazoline groups were also investigated since the design included quinazoline fragments with varying substitution patterns. HepG-2 cells were generally more sensitive to the tested compounds than MCF-7 cells. The type of linking group between two moieties, the imino, and the iminophenyl groups, and their effects on the cytotoxicity were the next area of investigation. It was also found that the two cell lines’ sensitivity to 2-phenyl quinazolines **18**–**24,** bearing the iminophenyl linker, was generally lower than that of quinazolines **7**–**13** that have the shorter imino linking group (Figure 3). A brief summary of the Structural activity relationship (SAR) analysis is shown in Figure 3.

#### 2.2.2. DNA-Flow Cytometry

**Figure 4:**
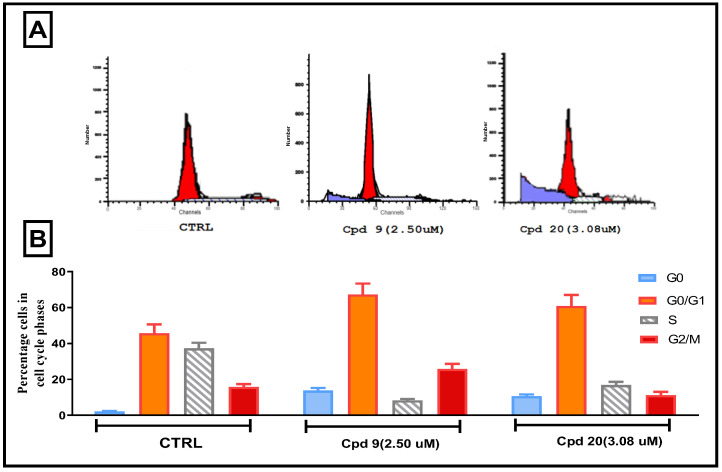
Effect of compounds **9** and **20** on the cell cycle distribution of HepG-2 cells after treatment of the cells with the mentioned compounds at their IC_50_ values. (**A**) DNA ploidy flow cytometric analysis. (**B**) The percentage of HepG-2 cells in the control and the treated cells at each stage of the cell cycle.

HepG-2 cells were treated with compounds **9** and **20** at their IC_50_ values in order to assess their effects on the cell cycle distribution (Figure 4). In this experiment, untreated cells served as a control group. The untreated cells showed the expected normal cell cycle distribution, with the majority of cells (45.22%) in the G0/G1 phase (Figure 4). Cells treated with compound **9** showed an increase in the G0/G1 phase (67.16%), which could suggest a partial arrest in this phase with a rise in the sub-G0/G1 phase (13.82%), indicating the induction of apoptosis. Compound **20** likewise had a comparable effect. There was a notable rise in the sub-G0/G1 phase (10.59%), indicating a strong activation of apoptosis. Interestingly, there was an increase in the G0/G1 phase (60.3%), suggesting possible alterations in cell cycle development. The changes observed in the cell cycle distribution of the treated cells by compounds **9** and compound **20** compared with the control cells highlight the significant cell cycle arrest at the G_1_/S phase together with the apoptosis-inducing activity of the tested compounds.

#### 2.2.3. Annexin-V-FITC/PI Assay

**Figure 5:**
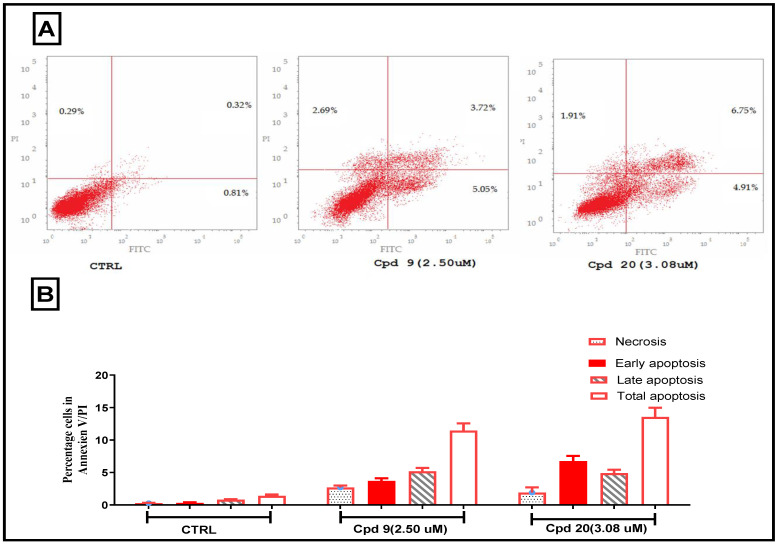
(**A**) Flow cytometry analysis of HepG2 cells double-stained with annexin-V FITC and propidium iodide (PI) following treatment with compounds **9** and **20** at their respective IC_50_ concentrations. The plots display the distribution of cells across different stages of apoptosis, enabling the assessment of early and late apoptotic effects induced by each compound. (**B**) Graphical representation of apoptotic cell population percentages showing both early and late apoptosis cell population percentages for the control cells and cells treated with compounds **9** and **20**.

To evaluate the activity of the tested compounds towards apoptosis induction, a fluorescent Annexin-V/propidium iodide (PI) staining assay was performed for compounds **9** and **20** at their IC_50_ values (Figure 5). Upon treatment of the cells with compounds **9** and **20**, an apparent increase in the percentage of cells within the early apoptotic stage was observed (Figure 5A,B) by around 6-fold compared with the untreated cell control. Moreover, the cell population at late apoptosis was increased upon treatment with compounds **9** and **20**, which were around 11- and 21-fold higher than the untreated control, respectively (Figure 5B). Both compounds **9** and **20** induced early and late apoptosis, demonstrating their abilities to trigger programmed cell death in the treated HepG2 cells.

#### 2.2.4. Kinase Enzyme Inhibitory Assays

**Figure 6:**
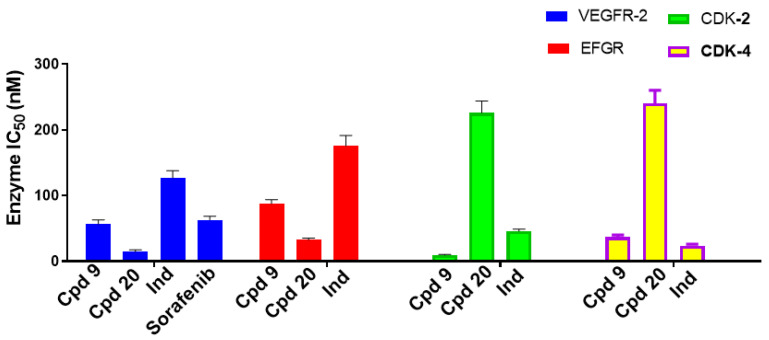
Graphical representation of the IC_50_ values (nM) of compounds **9** and **20** and Indirubin with VEGFR-2, EGFR, CDK-2, and CDK-4. The mean IC_50_ values are shown ± SEM. Data in the figure are associated with the data shown in Table 1.

To assess the kinase enzyme inhibitory effects, the most cytotoxic compounds (compounds **9** and **20**) from the in vitro cancer cell growth inhibition experiment were chosen. Each compound’s IC_50_ values were calculated against two CDK enzymes (CDK-2 and CDK-4) and two angiokinases (VEGFR-2 and EGFR) (Table 1 and Figure 6). For comparison, the reference compound, indirubin, was also assessed. Sorafenib was used as a positive control for only the VEGFR-2 enzyme assay with IC_50_ (63.27 nM). Indirubin showed expected moderate inhibition of all examined kinases. Compared to CDK-2, indirubin inhibited CDK-4 more potently (IC_50_ = 23.64 nM vs. 45.6 nM). With IC_50_ values of 56.74 nM and 9.39 nM, compound **9** demonstrated strong inhibition against VEGFR-2 and CDK-2, respectively (Table 1 and Figure 6). Multitargeted kinase inhibition may be possible given the dual inhibitory activity that has been demonstrated. Furthermore, compound **9** was found to exhibit kinase inhibitions that were greater than the inhibitory activity of indirubin over CDK enzymes. Compound **9** was around five times more potent than indirubin when it came to inhibiting CDK-2. The observed variations in potencies may be explained by the structural integration of an iminio group as a hydrogen bond acceptor, which could be responsible for the improved inhibitory impact observed with compound **9**. On the other hand, compound **20** demonstrated strong inhibition of both EGFR and VEGFR-2 with IC_50_ values of 14.31 nM and 32.65 nM, respectively (Table 1 and Figure 6). Compound **20** outperformed indirubin in terms of inhibitory action, with around 8.8- and 5.4-fold higher potency against VEGFR-2 and EGFR kinases, respectively. Additionally, compound **20** exhibited modest inhibitory activity against the CDK-2 and CDK-4 kinases. Significantly, the kinase enzyme inhibitory assays demonstrated a connection between the abilities of compound **9** and compound **20** to inhibit the main kinase enzymes under study and their observed cytotoxic activities.

### 2.3. ELISA Immunoassay

The p53 protein is known as a tumor suppressor protein and is also well known for its essential function in regulating cell cycle arrest and cell death. To perform its regulatory tasks, p53 controls downstream proteins including cyclin-dependent kinase (CDK) inhibitor p21. In addition, tumor growth is known to be inhibited by p21, which induces this effect by preventing CDK activation, leading to G1 phase arrest.

**Figure 7:**
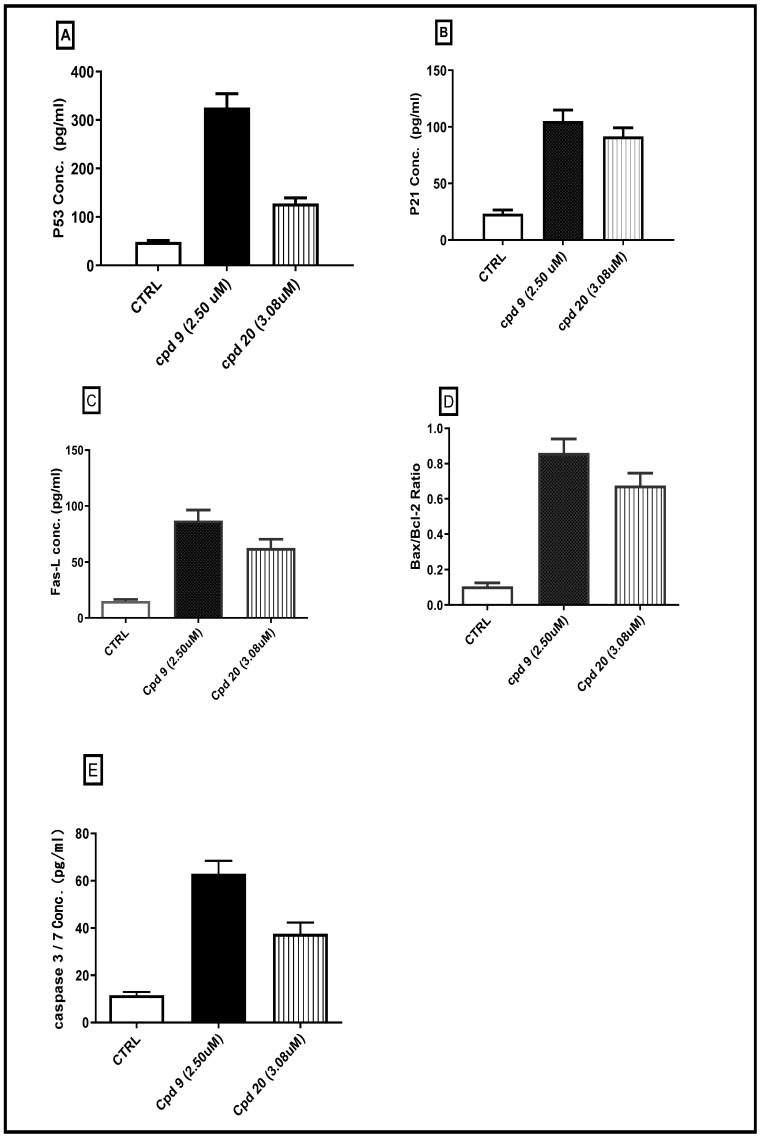
Elisa assay measurements showing the effects of compounds **9** and **20** on HepG2 cells at their IC_50_ (μM) compared to untreated cells as a negative control. Effects observed on levels of (**A**) p53, (**B**) p21, (**C**) Fas-L, (**D**) Bax/BCL-2 ratio, and (**E**) caspase 3/7. Data are expressed as the mean (*n* = 3 experiments) ± SEM.

In order to explain the effect of compounds **9** and **20** on cell cycle arrest, the compounds were tested with HepG-2 cells for their effects on expressed mediators, such as p53 and p21, which are involved in controlling the cell cycle. Upon treatment of HepG-2 cells with compound **9** at its IC_50_, a notable upregulation of p53 and p21 expression was observed by 6.7- and 4.52- fold, respectively, compared to untreated cells (Figure 7A,B). In a similar manner, compound **20** treatment resulted in considerable activation of p53 and p21 expression, where it increased the concentration of p53 and p21 by 2.65- and 3.9-fold compared with untreated cells. All of these findings point to the potential regulatory role of the p53 and p21 proteins in the observed cell cycle arrest of HepG-2 cells at the G1 phase.

Recent research has connected chemotherapy’s ability to induce cancer cell apoptosis with its efficacy. Cancer cells might undergo apoptosis through extrinsic or intrinsic pathways. An increase in the level of the tumor necrosis factor (TNF) family member Fas-L (CD95L) initiates the extrinsic death pathway. On the other hand, the intrinsic mechanism of apoptosis is triggered by mitochondrial activation and is controlled by upregulation of the Bax/Bcl-2 ratio. Moreover, It is interesting to note that these two different apoptotic signaling pathways combine when terminal caspases 3 and 7 are activated, causing cancer cells to undergo apoptosis [33].

To investigate the mechanisms underlying apoptosis in HepG-2 cells treated with the compounds **9** and **20**, we assessed the concentration of the following proteins: Fas-L, Bax, Bcl-2, and caspase 3/7. Compounds **9** and **20** significantly increased the levels of Fas-L expression, showing 5.7- and 4.1-fold increases, respectively, in comparison to the untreated control (Figure 7C). Turning to the mitochondrial apoptotic mediators, compounds **9** and **20** exhibited marked upregulation of the Bax/Bcl-2 ratio of 8.0- and 6.4-fold, respectively, by a concurrent increase in Bax concentration and downregulation of Bcl-2 concentration compared with the untreated cell control (Figure 7D). Meanwhile, compounds **9** and **20** increased caspase 3/7 levels by 5.3 -and 3.2-fold compared with untreated cells (Figure 7E). In conclusion, compounds **9** and **20** had an increased Bax/Bcl-2 ratio with a subsequent increase in caspase3/7 level. Collectively, the findings reported herein suggest that compounds **9** and **20** promote apoptosis through both intrinsic and extrinsic apoptotic pathways. This is due to their increased expression of Fas-L and higher Bax/Bcl-2 ratio.

### 2.4. Molecular Docking Investigations

Docking studies were performed with Molecular Operating Environment (MOE) [34] to explain the observed potencies in this study and investigate the binding interactions with different kinases. Compound **9** was docked into the binding site of CDK-2, and the observed binding interactions and binding energy were compared with indirubin, as a known CDK-2 inhibitor. Compound **20** was docked into the binding site of VEGFR-2, and the docking results were also compared with the VEGFR-2 inhibitor, sorafenib. The crystal structures of the wild-type proteins used in this study were selected based on several criteria, including their high resolution, well-defined active sites, and the presence of co-crystallized inhibitors structurally similar to the target compounds.

#### 2.4.1. Molecular Docking with the CDK-2 Binding Site

**Figure 8:**
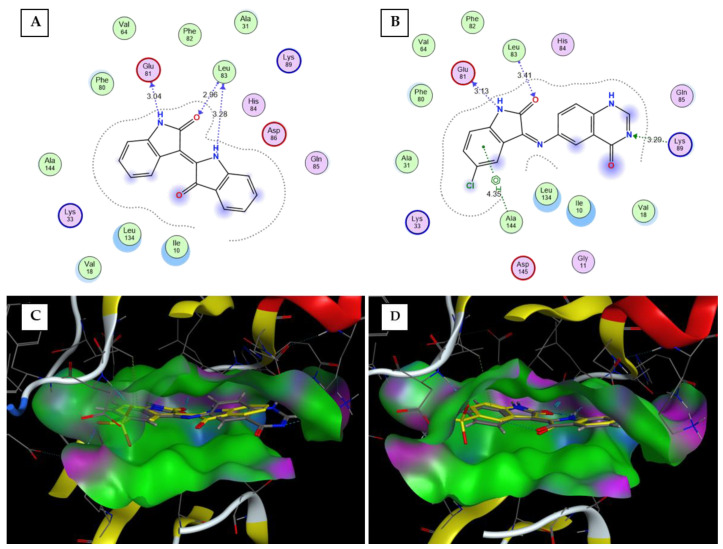
Docking poses displaying 2D interactions (Panels **A**,**B**) and 3D placement (Panels **C**,**D**) of indirubin and compound **9** within the CDK-2 active site. In (Panels **A**,**B**), hydrogen bonds are displayed as blue or green dotted arrows. The blue dotted arrows indicate the backbone donor/acceptor, while the green dotted arrows indicate the side chain donor/acceptor. The green dotted line indicates an arene-H interaction. Amino acid representations include spheres with the following color coding: red-outlined pink for acidic residues, blue-outlined pink for basic, black-outlined pink for polar, and black-outlined green for greasy amino acid residues. Binding distances between the docked compounds and active site residues (numbered in figures) are displayed in Angstroms. In (Panels **C**,**D**), the superimposition of the docked compound (grey) with the co-crystallized ligand (yellow) showing proper binding and placement of the docked compound in the active site of CDK-2 along with the same interactions displayed in (Panels **A**,**B**); the green surface indicates hydrophobic regions, purple marks hydrogen bond sites, and blue represents weakly polar areas. Atom color scheme: grey for carbon, red for oxygen, and blue for nitrogen.

To validate the docking steps, indirubin was first docked into the active site of CDK-2 (Figure 8A). The docked compound has good superimposition with the co-crystallized inhibitor, indirubin-5-sulphonate (Figure 8C). As expected, the three reported hydrogen bonding interactions between N1 of indirubin and Glu81, O2 of indirubin and Leu83, and N1’ of indirubin with Leu 83 were observed as the essential interactions for binding between indirubin and CDK-2 (Figure 8A) [35]. Upon docking of compound **9** into the CDK-2 active site, it formed two hydrogen bonding interactions between compound **9**’s indirubin ring N1 and O2 with Glu81 and Leu83, respectively (Figure 8B,D). Interestingly, two additional interactions were observed between the quinoline ring of compound **9** and the CDK-2 binding site. One of the interactions was a hydrogen bonding interaction between the N3 of the quinazolin-4-one ring and Lys89 (Figure 8B,D). The fourth interaction obtained was an arene-H interaction between the chloro-substituted aromatic ring of compound **9** with Ala144 of CDK-2 binding site. The observed additional interactions of compound **9** and specifically of the quinazoline ring suggest that the overall binding affinity of compound **9** is higher than that of indirubin and could be the reason behind the stronger inhibition of CDK-2 with compound **9** compared with indirubin.

#### 2.4.2. Molecular Docking with the Active Site of VEGFR-2

**Figure 9:**
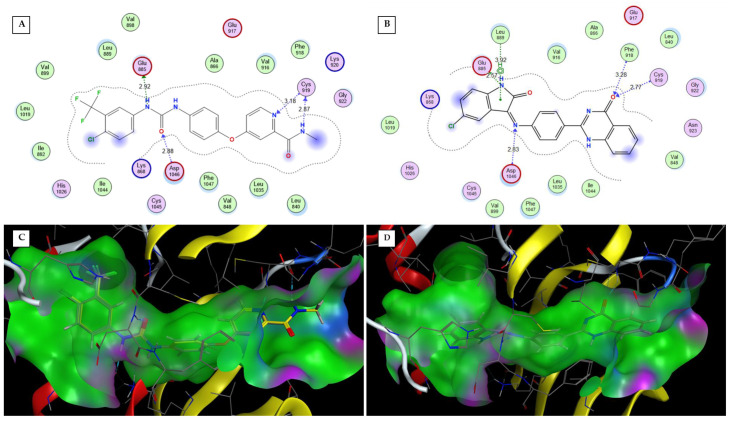
Docking poses displaying 2D interactions (**A**,**B**) of sorafenib and compound **20** and 3D placement (**C**) of compound **20** in the binding site of VEGFR-2. In (Panels **A**,**B**), Hydrogen bonds are displayed as blue or green dotted arrows. The blue dotted arrows indicate the backbone donor/acceptor, while the green dotted arrows indicate the side chain donor/acceptor. The green dotted line indicates an arene-H interaction. Amino acid representations include spheres with the following color coding: red-outlined pink for acidic residues, blue-outlined pink for basic, black-outlined pink for polar, and black-outlined green for greasy amino acid residues. (Panels **C**,**D**) show the docked compounds (grey) with the co-crystallized ligand (yellow, in panel **C**) and display the proper binding and placement of the docked compounds in the active site of VEGFR-2 along with the same interactions displayed in (Panels **A**,**B**); the green surface indicates hydrophobic regions, purple marks hydrogen bond sites, and blue represents weakly polar areas. Atom color scheme: grey for carbon, red for oxygen, blue for nitrogen, and yellow for sulfur. Binding distances between the docked compounds and active site residues (numbered in figures) are displayed in Angstroms. In (Panels **C**,**D**), the transparency of the front face of the binding site surface was increased in order to clearly show the bound compounds inside the active site of VEGFR-2.

To validate the docking parameters, sorafenib, the ligand bound to the VEGFR-2 crystal structure, was re-docked into the VEGFR-2 active site. As expected, the heterocyclic ring of sorafenib formed a hydrogen bonding interaction with Cys919, while the ureido group formed two hydrogen bonding interactions with Glu885 and Asp1046 within the regulatory domain pocket (RDP) (Figure 9A,B) [36]. With the validated docking parameters, compound **20** was docked into the binding site to evaluate its binding interactions and alignment within the active site. The quinalzoline ring of compound **20** showed two favorable interactions with the ATP binding site, including hydrogen bonds with Phe918 and Cys919, where it acted as a hydrogen bond acceptor (Figure 9B,D). Moreover, due to the nature of the quinazoline heteroaromatic ring, it could possibly have hydrophobic interactions with the greasy amino acids lining the ATP binding pocket (Figure 9D). Interestingly, the imino indolinone ring of compound **20** formed three interactions with the RDP amino acids. Two of the observed interactions were direct hydrogen bonds with Glu885, and Asp1046, while the third interaction observed was an arene-H interaction between the pyrrolone ring and Leu889 (Figure 9B). The results of the docking study suggest that compound **20** binds to VEGFR-2 as a type II kinase inhibitor similar to sorafenib where the phenyl ring spacer positions both the quinazoline ring and the indolinone favorably into the ATP binding pocket and into the regulatory domain pocket with a total of five favorable interactions with both pockets (Figure 9B,D).

## 3. Experimental

### 3.1. Materials and Instrumentation

Melting points were measured with a Stuart melting point apparatus and were reported as uncorrected values. NMR spectra were recorded by Varian Gemini-300BB 300 MHz FT-NMR spectrometers (Varian Inc., Palo Alto, CA, USA). ^1^HNMR and ^13^CNMR spectra were run at 300 and 75 MHz, respectively, in deuterated dimethylsulfoxide (DMSO-*d_6_*). Chemical shifts (*δ*_H_) were reported relative to TMS as an internal standard. All coupling constant (*J*) values are given in hertz. Chemical shifts (*δ*_C_) are reported relative to DMSO-*d_6_* as an internal standard. The abbreviations used are as follows: s, singlet; d, doublet; t, triplet; m, multiplet. IR spectra were recorded with a Bruker FT-IR spectrophotometer (Billerica, Massachusetts). Electron impact (EI) mass spectra were recorded on a Hewlett Packard 5988 spectrometer. Reactions and product mixtures were routinely monitored by thin layer chromatography (TLC) on silica gel precoated F_254_ Merck plates. A hexane–ethyl acetate (8.5:1.5 mL) mixture was used as a developing solvent system, and the spots were visualized by a UV lamp. Unless otherwise noted, all solvents and reagents were commercially available and used without further purification.

#### 3.1.1. Chemical Synthesis of Compounds **7**–**13** and **18**–**24**

The intermediate compounds 3H-Quinazolin-4-one (**2**) [22], 6-nitro-3H-quinazolin-4-one (**4**) [22,23,24], and 6-amino-3H-quinazolin-4-one (**5**) [25] were synthesized according to the reported procedure.

#### 3.1.2. General Procedure for the Synthesis of 6-(5-Substituted-2-oxo-1,2-dihydro-indol-3-ylideneamino)-3H-quinazolin-4-ones (**7**–**13**)

A solution of the proper isatin derivative (**6a**–**g**) (0.01 mol) and 6-amino-3H-quinazolin-4-one (**5**) (1.61 g, 0.01 mol) in glacial acetic acid (1 mL) and ethanol (10 mL) was heated under reflux for 4–6 h. The reaction was monitored with TLC for completion. The solvent was then evaporated under reduced pressure, and the produced solid was recrystallized from ethanol and afforded compounds **7**–**13**.

##### 6-(2-Oxo-1,2-dihydro-indol-3-ylideneamino)-3H-quinazolin-4-one (**7**)

Orange powder (2.05 g, 71%); m.p. 288–290 °C; Anal.Calcd. for C_16_H_10_N_4_O_2_ (290.28): C, 66.20; H, 3.47; N, 19.30; Found, C, 66.38; H, 3.53; N, 19.54; IR (KBr, cm^−1^): 3169 (NH), 3200 (NH), 1664 (C=O) 1685 (C=O); ^1^H-NMR (DMSO-*d*_6_) δ ppm: 6.39 (d, 1H, H-4 indolin-2-one, *J* = 7.2 Hz), 6.71 (overlapped dd, 1H, H-6 indolin-2-one), 6.91 (d, 1H, H-7 indolin-2-one, *J* = 7.2 Hz), 7.35 (overlapped dd, 1H, H-5 indolin-2-one), 7.50 (d, 1H, H-7 quinazoline, *J* = 8.7 Hz), 7.79 (d, 1H, H-8 quinazoline, *J* = 8.4 Hz), 8.03 (s, 1H, quinazoline), 8.08 (s, 1H, quinazoline), 10.99 (s, 1H, NH, D_2_O exchangeable), 12.23 (s, 1H, NH, D_2_O exchangeable); ^13^C-NMR (DMSO-*d*_6_) δ ppm: 113.6, 115.2, 124.0, 125.6, 127.2, 127.9, 129.6, 144.1, 144.7, 145.3, 146.9, 147.6, 148.4, 154.1, 161.1, 163.9; MS *m*/*z* [%]: 290 [M^+^, 4.11], 161 [100].

##### 6-(5-Flouro-2-oxo-1,2-dihydro-indol-3-ylideneamino)-3H-quinazolin-4-one (**8**)

Red powder (1.69 g, 55%); m.p. 290–293 °C; Anal.Calcd. for C_16_H_9_FN_4_O_2_ (308.27): C, 62.34; H, 2.94; N, 18.17; Found, C, 62.63; H, 2.91; N, 18.45; IR (KBr, cm^−1^): 3199 (NH), 3250 (NH), 1665 (C=O), 1687 (C=O); ^1^H-NMR (DMSO-*d*_6_) δ ppm: 6.11 (d, 1H, H-4 indolin-2-one, *J* = 8.4 Hz), 6.90–6.94 (m, 2H, indolin-2-one), 7.52 (d, 1H, H-7 quinazoline, *J* = 8.4 Hz), 7.81 (d, 1H, H-8 quinazoline, *J* = 8.7 Hz), 8.02 (s, 1H, quinazoline), 8.08 (s, 1H, quinazoline), 10.98 (s, 1H, NH, D_2_O exchangeable), 12.25 (s, 1H, NH, D_2_O exchangeable); ^13^C-NMR (DMSO-*d*_6_) δ ppm: 112.4, 113.6, 115.2, 121.7, 124.0, 125.6, 127.2, 127.9, 129.5, 144.1, 145.3, 146.9, 147.6, 148.4, 161.1, 163.8.

##### 6-(5-Chloro-2-oxo-1,2-dihydro-indol-3-ylideneamino)-3H-quinazolin-4-one (**9**)

Orange-red powder (1.19 g, 59%); m.p. 292–294 °C; Anal.Calcd. for C_16_H_9_ClN_4_O_2_ (324.72): C, 59.18; H, 2.79; N, 17.25; Found, C, 59.42; H, 2.81; N, 17.51; IR (KBr, cm^−1^): 3163 (NH), 3210 (NH), 1675 (C=O), 1694 (C=O); ^1^H-NMR (DMSO-*d*_6_) δ ppm: 6.31 (s, 1H, H-4 indolin-2-one), 6.95 (d, 1H, indolin-2-one, *J* = 8.7 Hz), 7.43 (d, 1H, indolin-2-one, *J* = 8.7 Hz), 7.53 (d, 1H, H-7 quinazoline, *J* = 8.1 Hz), 7.82 (d, 1H, H-8 quinazoline, *J* = 8.4 Hz), 8.04 (s, 1H, quinazoline), 8.10 (s, 1H, quinazoline), 11.11 (s, 1H, NH, D_2_O exchangeable), 12.33 (s, 1H, NH, D_2_O exchangeable); ^13^C-NMR (DMSO-*d*_6_) δ ppm: 113.7, 115.3, 117.3, 123.0, 125.6, 126.9, 127.3, 127.9, 129.5, 134.2, 146.5, 147.6, 148.5, 155.2, 161.0, 163.5; MS *m*/*z* [%]: 326 [(M + 2)^+^, 1.48], 324 [M^+^, 4.45], 260 [100].

##### 6-(5-Bromo-2-oxo-1,2-dihydro-indol-3-ylideneamino)-3H-quinazolin-4-one (**10**)

Brown powder (2.58 g, 70%); m.p. >300 °C; Anal.Calcd. for C_16_H_9_BrN_4_O_2_ (369.18): C, 52.06; H, 2.46; N, 15.18; Found, C, 52.21; H, 2.49; N, 15.37; IR (KBr, cm^−1^): 3175 (NH), 3222 (NH), 1650 (C=O), 1694 (C=O); ^1^H-NMR (DMSO-*d*_6_) δ ppm: 6.44 (s, 1H, H-4 indolin-2-one), 6.87 (d, 1H, indolin-2-one, *J* = 8.7 Hz), 7.65–7.67 (m, 2H, indolin-2-one and quinazoline), 7.79 (d, 1H, H-8 quinazoline, *J* = 8.4 Hz), 8.04 (s, 1H, quinazoline), 8.10 (s, 1H, quinazoline), 11.13 (s, 1H, NH, D_2_O exchangeable), 12.33 (s, 1H, NH, D_2_O exchangeable); ^13^C-NMR (DMSO-*d*_6_) δ ppm: 111.7, 113.6, 115.6, 117.8, 120.3, 121.9, 122.7, 123.5, 130.3, 142.5, 145.0, 147.6, 152.4, 154.1, 160.4, 163.6; MS *m*/*z* [%]: 370 [(M + 2)^+^, 4.58], 368 [M^+^, 6.11], 161 [100].

##### 6-(5-Nitro-2-oxo-1,2-dihydro-indol-3-ylideneamino)-3H-quinazolin-4-one (**11**)

Yellow powder (2.14 g, 64%); m.p. 298–300 °C; Anal.Calcd. for C_16_H_9_N_5_O_4_ (335.28): C, 57.32; H, 2.71; N, 20.89; Found, C, 57.48; H, 2.70; N, 21.13; IR (KBr, cm^−1^): 3166 (NH), 3258 (NH), 1658 (C=O), 1668 (C=O); ^1^H-NMR (DMSO-*d*_6_) δ ppm: 6.94 (d, 1H, H-4 indolin-2-one, *J* = 9 Hz), 7.33 (d, 1H, indolin-2-one, *J* = 9 Hz), 7.66–7.69 (m, 3H, indolin-2-one and quinazoline), 8.00 (s, 1H, quinazoline), 8.02 (s, 1H, quinazoline), 10.31 (s, 1H, NH, D_2_O exchangeable),10.91 (s, 1H, NH, D_2_O exchangeable);^13^C-NMR (DMSO-*d*_6_) δ ppm: 107.6, 115.1, 119.6, 121.6, 127.1, 129.2, 131.6, 135.9, 136.0, 138.0, 148.7, 151.8, 156.3, 157.1, 161.5, 163.5; MS m/z [%]: 335 [M+, 0.79], 42 [100].

##### 6-(5-Methyl-2-oxo-1,2-dihydro-indol-3-ylideneamino)-3H-quinazolin-4-one (**12**)

Brown powder (1.45 g, 48%); m.p. >300 °C; Anal.Calcd. for C_17_H_12_N_4_O_2_ (304.31): C, 67.10; H, 3.97; N, 18.41; Found, C, 67.32; H, 4.02; N, 18.59; IR (KBr, cm^−1^): 3185 (NH), 3248 (NH), 1666 (C=O), 1683 (C=O); ^1^H-NMR (DMSO-*d*_6_) δ ppm: 2.09 (s, 3H, CH_3_ of indolin-2-one), 6.80 (s, 1H, H-4 indolin-2-one), 7.26 (d, 1H, indolin-2-one, *J* = 6 Hz), 7.52 (d, 1H, indolin-2-one, *J* = 8.7 Hz), 7.71 (d, 1H, H-7 quinazoline, *J* = 8.4 Hz), 7.96 (d, 1H, H-8 quinazoline, *J* = 9 Hz), 8.45 (s, 1H, quinazoline), 8.60 (s, 1H, quinazoline), 10.30 (s, 1H, NH, D_2_O exchangeable), 10.99 (s, 1H, NH, D_2_O exchangeable). ^13^C-NMR (DMSO-*d*_6_) δ ppm: 22.2, 116.8, 118.1, 119.7, 123.0, 123.5, 128.8, 129.3, 131.3, 136.4, 136.9, 138.6, 146.6, 151.4, 152.3, 161.4, 163.2.

##### 6-(5-Methoxy-2-oxo-1,2-dihydro-indol-3-ylideneamino)-3H-quinazolin-4-one (**13**)

Brown powder (1.60 g, 50%); m.p. >300 °C; Anal.Calcd. for C_17_H_12_N_4_O_3_ (320.31): C, 63.75; H, 3.78; N, 17.49; Found, C, 63.91; H, 3.75; N, 17.73; IR (KBr, cm^−1^): 3154 (NH), 3234 (NH), 1675 (C=O), 1685 (C=O); ^1^H-NMR (DMSO-*d*_6_) δ ppm: 3.41 (s, 3H, OCH_3_ of indolin-2-one), 6.80 (s, 1H, H-4 indolin-2-one), 6.99 (d, 1H, indolin-2-one, *J* = 6.6 Hz), 7.49–7.52 (m, 2H, indolin-2-one and quinazoline), 7.81 (d, 1H, H-8 quinazoline, *J* = 9 Hz), 8.14 (s, 1H, quinazoline), 8.34 (s, 1H, quinazoline), 10.30 (s, 1H, NH, D_2_O exchangeable), 10.75 (s, 1H, NH, D_2_O exchangeable). ^13^C-NMR (DMSO-*d*_6_) δ ppm: 56.6, 113.6, 115.2, 121.5, 121.8, 124.0, 125.6, 127.2, 127.9, 129.6, 144.1, 145.3, 146.9, 147.6, 148.2, 161.1, 163.9.

#### 3.1.3. Synthesis of the Intermediates 2-(4-Nitro-phenyl)-3H-quinazolin-4-one (**16**) and 2-(4-Aminophenyl)quinazolin-4(3H)-one (**17**)

Compound (**16**) [26] and (**17**) [27] were synthesized according to the reported procedure.

#### 3.1.4. General Procedure for the Synthesis of 2-[4-(5-Substituted-2-oxo-1,2-dihydro-indol-3-ylideneamino)-phenyl]-3H-quinazolin-4-one (**18**–**24**)

A solution of the isatin derivative (**6a**–**g**) (0.01 mol), 2-(4-aminophenyl)quinazolin-4(3H)-one (**17**) (2.37 g, 0.01 mol)o and glacial acetic acid (1 mL) in ethanol (10 mL) was heated under reflux for 6 h. The solvent was evaporated under reduced pressure and the residue formed was crystallized from ethanol to afford compounds **18**–**24**.

##### 2-[4-(2-Oxo-1,2-dihydro-indol-3-ylideneamino)-phenyl]-3H-quinazolin-4-one (**18**)

Orange-yellow powder (3.29 g, 45%); m.p. >300 °C; Anal.Calcd. for C_22_H_14_N_4_O_2_ (366.37): C, 72.12; H, 3.85; N, 15.29; Found, C, 72.41; H, 3.43; N, 14.72; IR (KBr, cm^−1^): 3142 (NH), 3250 (NH), 1665 (C=O), 1666 (C=O); ^1^H-NMR (DMSO-*d*_6_) δ ppm: 6.48 (d, 1H, H-4 indolin-2-one, *J* = 9 Hz), 6.74 (overlapped dd, 1H, H-6 indolin-2-one), 6.93 (d, 1H, H-7 indolin-2-one, *J* = 9 Hz), 7.17 (d, 2H, Ar-H, *J* = 8.4 Hz), 7.36 (overlapped dd, 1H, H-5 indolin-2-one), 7.55 (overlapped dd, 1H, quniazoline), 7.75 (d, 1H, quinazoline, *J* = 7.5 Hz), 7.83 (overlapped dd, 1H, quinazoline), 8.17 (d, 1H, quinazoline, *J* = 7.2 Hz), 8.33 (d, 2H, Ar-H, *J* = 8.7 Hz), 10.94 (s, 1H, NH, D_2_O exchangeable), 12.46 (s, 1H, NH, D_2_O exchangeable); ^13^C-NMR (DMSO-*d*_6_) δ ppm: 115.5, 117.4, 117.5, 120.8, 121.8, 125.5, 125.8, 127.4, 128.1, 134.5, 141.2, 144.0, 147.1, 148.7, 151.7, 153.2, 155.0, 162.1, 163.2; MS *m*/*z* [%]: 366 [M^+^, 20.35], 119 [100].

##### 2-[4-(5-Flouro-2-oxo-1,2-dihydro-indol-3-ylideneamino)-phenyl]-3H-quinazolin-4-one (**19**)

Dark red powder (3.91 g, 51%); m.p. >300 °C; Anal.Calcd. for C_22_H_13_FN_4_O_2_ (384.36): C, 68.75; H, 3.41; N, 14.58; Found, C, 68.98; H, 3.45; N, 14.90; IR (KBr, cm^−1^): 3175 (NH), 3245 (NH), 1663 (C=O), 1665 (C=O); ^1^H-NMR (DMSO-*d*_6_) δ ppm: 6.13 (d, 1H, H-4 indolin-2-one, *J* = 7 Hz), 6.93 (d, 1H, H-6 indolin-2-one, *J* = 7 Hz), 7.16–7.19 (m, 3H, H-7 indolin-2-one and Ar-H), 7.53 (overlapped dd, 1H, quinazoline), 7.76 (d, 1H, quniazoline, *J* = 7.5 Hz), 7.83 (overlapped dd, 1H, quinazoline), 8.16 (d, 1H, quinazoline, *J* = 7.8 Hz), 8.33 (d, 2H, Ar-H, *J* = 8.4 Hz), 10.93 (s, 1H, NH, D_2_O exchangeable), 12.55 (s, 1H, NH, D_2_O exchangeable). ^13^C-NMR (DMSO-*d*_6_) δ ppm: 113.7, 115.3, 117.3, 123.9, 124.9, 125.6, 126.9, 127.2, 127.9, 129.5, 134.2, 142.3, 145.3, 146.4, 146.9, 147.5, 148.4, 155.2, 158.8, 160.9, 163.5; MS *m*/*z* [%]: 384 [M^+^, 14], 84 [100].

##### 2-[4-(5-Chloro-2-oxo-1,2-dihydro-indol-3-ylideneamino)-phenyl]-3H-quinazolin-4-one (**20**)

Orange powder (4.88 g, 61%); m.p. >300 °C; Anal.Calcd. for C_22_H_13_ClN_4_O_2_ (400.82): C, 65.92; H, 3.27; N, 13.98; Found, C, 66.18; H, 3.30; N, 14.21; IR (KBr, cm^−1^):3173 (NH), 3230 (NH), 1670 (C=O), 1672 (C=O); ^1^H-NMR (DMSO-*d*_6_) δ ppm: 6.39 (s, 1H, H-4 indolin-2-one), 6.95 (d, 1H, H-6 indolin-2-one, *J* = 8.7 Hz), 7.15–7.20 (m, 3H, H-7 indolin-2-one and Ar-H), 7.52 (overlapped dd, 1H, quinazoline), 7.76 (d, 1H, quniazoline, *J* = 7.8 Hz), 7.83 (overlapped dd, 1H, quinazoline), 8.17 (d, 1H, quinazoline, *J* = 6.3 Hz), 8.35 (d, 2H, Ar-H, *J* = 8.7 Hz), 11.13 (s, 1H, NH, D_2_O exchangeable), 12.54 (s, 1H, NH, D_2_O exchangeable); ^13^C-NMR (DMSO-*d*_6_) δ ppm: 114.9, 116.7, 117.4, 117.5, 120.9, 125.3, 125.8, 128.0, 129.3, 129.8, 134.5, 140.7, 144.5, 145.9, 148.7, 151.6, 152.5, 154.0, 160.1, 162.9. MS *m*/*z* [%]: 366 [M^+^, 20.35], 119 [100].

##### 2-[4-(5-Bromo-2-oxo-1,2-dihydro-indol-3-ylideneamino)-phenyl]-3H-quinazolin-4-one (**21**)

Dark brown powder (4.71 g, 53%); m.p. >300 °C; Anal.Calcd. for C_22_H_13_BrN_4_O_2_ (445.27): C, 59.34; H, 2.94; N, 12.58;Found, C, 59.62; H, 2.92; N, 12.78; IR (KBr, cm^−1^): 3175 (NH), 3242 (NH), 1666 (C=O), 1667 (C=O); ^1^H-NMR (DMSO-*d*_6_) δ ppm: 6.54 (s, 1H, H-4 indolin-2-one), 6.91 (d, 1H, H-6 indolin-2-one, *J* = 8.4 Hz), 7.16–7.20 (m, 3H, H-7 indolin-2-one and Ar-H), 7.57 (overlapped dd, 1H, quinazoline), 7.77 (d, 1H, quniazoline, *J* = 8.7 Hz), 7.83 (overlapped dd, 1H, quinazoline), 8.17 (d, 1H, quinazoline, *J* = 7.2 Hz), 8.36 (d, 2H, Ar-H, *J* = 8.7 Hz), 11.11 (s, 1H, NH, D_2_O exchangeable), 12.49 (s, 1H, NH, D_2_O exchangeable). ^13^C-NMR (DMSO-*d*_6_) δ ppm: 114.4, 117.3, 117.8, 120.7, 121.3, 126.5, 129.0, 131.3, 134.6, 138.2, 138.6, 144.3, 145.8, 148.8, 148.9, 152.1, 154.2, 157.9, 160.9, 162.3. MS *m*/*z* [%]: 446 [(M + 2)^+^, 0.98], 444 [M^+^, 0.97], 57 [100].

##### 2-[4-(5-Nitro-2-oxo-1,2-dihydro-indol-3-ylideneamino)-phenyl]-3H-quinazolin-4-one (**22**)

Yellow powder (3.86 g, 47%); m.p. >300 °C; Anal.Calcd. for C_22_H_13_N_5_O_4_ (411.37): C, 64.23; H, 3.19; N, 17.02, Found, C, 64.35; H, 3.23; N, 17.16; IR (KBr, cm^−1^): 3148 (NH), 3242 (NH), 1666 (C=O), 1668 (C=O); ^1^H-NMR (DMSO-*d*_6_) δ ppm: 7.12–7.19 (m, 1H, indolin-2-one), 7.26–7.30 (m, 4H, H-6 indolin-2-one, H-7 indolin-2-one and Ar-H), 7.54 (overlapped dd, 1H, quinazoline), 7.78 (d, 1H, quniazoline, *J* = 8.7 Hz), 7.84 (overlapped dd, 1H, quinazoline), 8.30 (d, 1H, quinazoline, *J* = 9.3 Hz), 8.40 (d, 2H, Ar-H, *J* = 8.7 Hz), 11.72 (s, 1H, NH, D_2_O exchangeable), 12.60 (s, 1H, NH, D_2_O exchangeable). ^13^C-NMR (DMSO-*d*_6_) δ ppm: 115.2, 119.0, 122.9, 123.0, 129.1, 129.2, 131.3, 132.4, 136.0, 136.2, 136.9, 142.9, 144.8, 146.1, 149.1, 152.2, 154.6, 156.2, 160.9, 162.4; MS *m*/*z* [%]: 366 [M^+^, 20.35], 119 [100].

##### 2-[4-(5-Methyl-2-oxo-1,2-dihydro-indol-3-ylideneamino)-phenyl]-3H-quinazolin-4-one (**23**)

Brown powder (4.56 g, 60%); m.p. >300 °C; Anal.Calcd. for C_23_H_16_N_4_O_2_ (380.40): C, 72.62; H, 4.24; N, 14.73; Found, C, 72.89; H, 4.31; N, 14.98; IR (KBr, cm^−1^): 3166 (NH), 3250 (NH), 1664 (C=O). 1666 (C=O); ^1^H-NMR (DMSO-*d*_6_) δ ppm: 2.26 (s, 3H, CH_3_), 6.31 (s, 1H, H-4 indolin-2-one), 6.82 (d, 1H, H-6 indolin-2-one, *J* = 8.1 Hz), 7.14–7.20 (m, 3H, H-7 indolin-2-one and Ar-H), 7.54 (overlapped dd, 1H, quinazoline, *J* = 7.2 Hz), 7.76 (d, 1H, quniazoline, *J* = 7.8 Hz), 7.84 (overlapped dd, 1H, quinazoline, *J* = 7.2 Hz), 8.17 (d, 1H, quinazoline, *J* = 6.9 Hz), 8.33 (d, 2H, Ar-H, *J* = 9 Hz), 10.85 (s, 1H, NH, D_2_O exchangeable), 12.46 (s, 1H, NH, D_2_O exchangeable). ^13^C-NMR (DMSO-*d*_6_) δ ppm: 20.3, 116.6, 117.5, 119.0, 121.7, 127.0, 127.2, 129.1, 129.2, 132.4, 135.9, 136.0, 138.0, 138.2, 143.3, 144.1, 148.7, 151.2, 152.0, 160.7, 162.5. MS *m*/*z* [%]: 366 [M^+^, 20.35], 119 [100].

##### 2-[4-(5-Methoxy-2-oxo-1,2-dihydro-indol-3-ylideneamino)-phenyl]-3H-quinazolin-4-one (**24**)

Brown powder (4.59 g, 58%); m.p. >300 °C; Anal.Calcd. for C_23_H_16_N_4_O_3_ (396.40): C, 69.69; H, 4.07; N, 14.13; Found, C, 69.93; H, 4.13; N, 14.35; IR (KBr, ν cm^−1^): 3165 (NH), 3240 (NH), 1660 (C=O), 1661 (C=O); ^1^H-NMR (DMSO-*d*_6_) δ ppm: 3.77 (s, 3H, OCH_3_), 5.94 (s, 1H, H-4 indolin-2-one), 6.84 (d, 1H, H-6 indolin-2-one, *J* = 9 Hz), 6.99 (d, 1H, H-7 indolin-2-one, *J* = 9 Hz), 7.17 (d, 2H, Ar-H, *J* = 9 Hz), 7.52 (overlapped dd, 1H, quinazoline,), 7.75 (d, 1H, quniazoline, *J* = 6 Hz), 7.83 (overlapped dd, 1H, quinazoline), 8.15 (d, 1H, quinazoline, *J* = 6 Hz), 8.32 (d, 2H, Ar-H, *J* = 9 Hz), 10.80 (s, 1H, NH, D_2_O exchangeable), 12.54 (s, 1H, NH, D_2_O exchangeable). ^13^C-NMR (DMSO-*d*_6_) δ ppm: 55.1, 113.9, 117.5, 120.5, 121.6, 127.0, 127.1, 129.1, 129.2, 131.7, 136.0, 138.2, 142.3, 143.1, 148.7, 151.2, 151.8, 152.0, 155.3, 160.1, 163.7. MS *m*/*z* [%]: 366 [M^+^, 20.35], 119 [100].

### 3.2. Biological Evaluation

#### 3.2.1. In Vitro Cancer Cell Growth Inhibition

##### Materials

Cytotoxic activity of the synthesized compounds was evaluated against HepG-2 and MCF-7 cancer cell lines using the MTT assay. The tested cell lines were obtained from the American Type Culture Collection (ATCC, Alexandria, MN, USA) through the Tissue Culture Unit, The Egyptian Organization for Biological Products and Vaccines (Vacsera, Giza, Egypt). Indirubin, chemicals, and solvents were purchased from Sigma-Aldrich (St. Louis, MO, USA).

##### Methodology

The cells were grown on RPMI-1640 medium supplemented with 10% inactivated fetal calf serum and 50 µg/mL gentamycin. The cells were maintained at 37 °C in a humidified atmosphere with 5% CO_2_ and were sub-cultured two to three times per week. For antitumor assays, the tumor cell lines were suspended in the medium at concentration 5 × 10^4^ cell/well in Corning^®^ 96-well tissue culture plates (Corning, Corning, NY, USA), then incubated for 24 h. The tested molecules were then added to 96-well plates (six replicates) to achieve six concentrations for each compound. Six vehicle controls with media or 0.5% DMSO were run for each 96-well plate as a control. After incubating for 24 h, the numbers of viable cells were determined by the MTT test. Briefly, the media was removed from the 96 well plate and replaced with 100 µL of fresh culture RPMI 1640 medium without phenol red then 10 µL of the 12 mM MTT stock solution (5 mg of MTT in 1 mL of PBS) to each well including the untreated controls. The 96-well plates were then incubated at 37 °C and 5% CO_2_ for 4 h. An 85 µL aliquot of the media was removed from the wells, and 50 µL of DMSO was added to each well, mixed thoroughly with the pipette, and incubated at 37 °C for 10 min. Then, the optical density was measured at 590 nm with the microplate reader (SunRise, TECAN, Inc., Morrisville, NC, USA) to determine the number of viable cells, and the percentage of viability was calculated as [1 − (ODt/ODc)] × 100%, where ODt is the mean optical density of wells treated with the tested sample and ODc is the mean optical density of untreated cells [37]. The relation between surviving cells and drug concentration was plotted to obtain the survival curve of each tumor cell line after treatment with the specified compound. The 50% inhibitory concentration (IC_50_), the concentration required to cause toxic effects in 50% of intact cells, was estimated from graphic plots of the dose–response curve for each conc. using GraphPad Prism software 10.4.0. The results obtained were compared with those of indirubin as the standard. Each concentration was repeated four times. The data associated with this experiment are reported in Table 1, Tables S1 and S2.

#### 3.2.2. Kinase Enzyme Inhibitory Assays

##### Materials

The enzyme inhibition assay for the tested molecules was performed at BPS Bioscience Corporation, San Diego, CA, USA. The following materials served as the enzyme sources, Poly (Glu, Tyr) sodium salt (4:1, Glu:Tyr) (Sigma#P7244) served as the standardized substrate, and the Kinase-Glo Plus Luminescence kinase assay kit (Promega # V3772) was used. The following enzyme kits were used: VEGFR-2 [BPS#40301], EGFR [BPS#40721], CDK2 [BPS#41101] and CDK-4 [BPS#79674] Glo Plus luminescence kinase assay kits. The assays were performed using Kinase-Glo Plus luminescence (Promega Corporation, Madison, WI, USA).

##### Methodology

The tested molecules 9, 20, and indurbin were diluted to 100 µL in DMSO (10%), and 5 µL of the solution was added to a 50-µL enzyme reaction until the final concentration of DMSO was 1% in all of reactions. The enzymatic reactions were conducted at 30 °C for 40 min. The reaction mixture contained enzyme substrate and respective kinases. After the enzymatic reaction, 50 µL of enzyme-Glo Plus luminescence topoisomerase assay solution (Promega) was added to each reaction, and the plate was incubated for 5 min at room temperature. The luminescence signal was measured using a BioTek Synergy 2 microplate reader. The luminescence data were analyzed using the computer software GraphPad Prism. The difference between luminescence intensities in the absence of enzyme (Lu_t_) and in the presence of enzyme (Lu_c_) was defined as 100% activity (Lu_t_ − Lu_c_). The values of enzyme activity percentages versus five concentrations of the small molecule were then plotted using a non-linear regression analysis of the sigmoidal dose–response curve. The IC_50_ value was determined using GraphPad Prism software. All experiments were repeated three times (Table S3).

Compounds **9** and **20** were selected to be evaluated against VEGFR-2, EGFR, CDK-2, and CDK-4 enzymes for the determination of their IC_50_ values compared with indirubin as a reference. The Kinase-Glo Plus luminescence kinase assay kit (Promega—V3772), Poly (Glu, Tyr) sodium salt, (4:1, Glu:Tyr) (Sigma—P7244), and VEGFR-2 [BPS#40301], EGFR [BPS#40721], CDK2 [BPS#41101] and CDK-4 [BPS#79674] Glo Plus luminescence kinase assay kits were used in these assays. The assay was done at Bioscience Laboratory BPS, California, USA. The assay measures kinase activity by quantitating the amount of ATP remaining in the solution following kinase reaction. The luminescent signal from the assay was correlated with the amount of ATP present and inversely correlated with kinase activity. The selected compounds **9** and **20** were diluted in 10% DMSO, and 5 µL of the diluted solutions were added to a 45-µL reaction so that the final concentration of DMSO was 1% in all reactions. All of the enzymatic reactions were conducted at 30 °C for 40 min. The reaction mixture contained 40 mM Tris, pH 7.4, 10 mM MgCl_2_, 0.1 mg/mL BSA, 1 mM DTT, 10 µM ATP, Kinase substrate, and VEGFR-2, EFGR, CDK-2 and CDK-4 enzymes. After the enzymatic reaction, 50 µL of Kinase-Glo Plus Luminescence kinase assay solution (Promega) was added to each reaction, and the plate was incubated for 5 min at room temperature. The luminescence signal was measured using a BioTek Synergy 2 microplate reader [38]. Kinase activity assays were performed in triplicate for each concentration. The luminescence data were analyzed using the Graphpad Prism software.

#### 3.2.3. DNA Flow Cytometry Analysis

The HepG-2 cells were incubated at a density of 3 × 10^6^ cells/mL RPMI-1640 medium in T-75 flasks for 24 h and then treated with tested molecules 9 and 20 at their IC_50_ (µM) for 24 h. The HepG-2 cells were then collected by trypsinization, washed with PBS, and fixed. After that, treated cells were stained using the Cycle Test Plus DNA Reagent Kit (BD Biosciences, San Jose, CA, USA) according to the manufacturer’s instructions [39]. The percentage of cell cycle distribution was calculated using CELLQUEST software 5.1 (Becton Dickinson Immuno-cytometry Systems, San Jose, CA, USA) (Table S4).

#### 3.2.4. Annexin Staining Apoptosis Analysis

HepG2 cells were cultured in a 10-cm plate for 24 h, after which IC_50_ concentrations of tested molecules 9 and 20 were added and incubated for 24 h. Then, the cells were washed with PBS and detached by trypsin. The detached cells were collected into a 15-mL centrifuged tube, washed with ice-cold PBS twice, and centrifuged at 1200 rpm for 5 min. A volume of 0.1 mL binding buffer was added to the treated HepG-2 cells, followed by adding 5 μL of annexin V-FITC and 5 μL of 50 μg/mL PI staining reagents. After mixing and reacting at 25 °C for 15 min in the dark, the apoptotic cell percentages were analyzed by a flow cytometer [FACS Calibur flow cytometer (BD Biosciences)] (Table S5).

#### 3.2.5. ELISA Assays for Cell Death Modulators and Apoptotic Biomarkers

HepG2 cells were cultured in RPMI 10% + 5%FBS penicillin/streptomycin and were incubated at a humidity 5% CO_2_ and maintained at 37 °C. HepG2 cells were treated with the IC_50_ concentration of compounds **9** and **20** (2.50 and 3.06 µM) for 24 h. HepG2 cells were cultured as a monolayer in T-25 flasks and were seeded to attain 30% confluency prior to treatment. The main 100 mM stock dissolved in DMSO was diluted with the cell culture medium in order to reach the previously determined IC_50_ concentration of each of the three preparations. After that, HepG2 cells were collected via trypsinization and centrifuged at 10,000 rpm. The pellet was then rinsed with PBS and lysed in RIPA lysis buffer at 4 °C for 45 min, then centrifuged at 14,000 rpm for 20 min to remove the cellular debris. Lysates were then collected and stored at 80 °C for protein determination. The levels of p53, p21, Fas-L, Bax, Bcl-2, and caspase 3/7 were assessed using the human P53 ELISA kit (Catalog No. ABIN6962151), P21 ELISA kit (Catalog No. ABIN6966175), Human Apoptosis Ligand (FAS-L), Bax ELISA kit, Human Factor-Related. Bcl-2 ELISA kit ELISA kit, and caspase3/7 ELISA kit (Elabscience Biotechnology Co., Wuhan, Hubei, China). The procedure of the used kits was done according to the manufacturer’s instructions. Briefly, equal amounts of cell lysates were loaded and then probed with specific antibodies. The samples were measured at 450 nm in ROBONEK P2000 ELISA reader [40]. The analysis was confirmed with three different sets of extracts. All experiments were done in triplicate (Tables S6–S9).

### 3.3. Molecular Docking Studies

Molecular Operating Environment (MOE program 2020.09; Chemical Computing Group, Montreal, QC, Canada) was used to perform the docking studies. Crystal structures of CDK-2 co-crystallized with indirubin-5-sulphonate inhibitor (PDB code 1E9H) [35], and of VGEFR-2 co-crystallized with sorafenib (PDB code 4ASD) [36] were downloaded from the RCSB protein data bank. The crystal structures were prepared for the molecular docking simulations by deleting unnecessary bound ligands, ions, and metals and by adding the missing protons using MOE software 2020.09. In CDK-2, chain B was deleted, and chain A was kept and utilized in the docking study. The docking parameters used with CDK-2 were the default triangle matcher for placement and London dG for initial scoring with a maximum number of 30 retained poses. Rigid receptor was used in the refinement with GBVI/WSA dG for final scoring, and 5 poses were retained. For VEGFR-2, the docking placement methodology included a pharmacophore placement with London dG as an initial scoring method with 30 retained poses. A post-placement refinement was then done with GBVI/WSA dG for scoring and 10 poses were retained. In each study, the best poses in terms of the number of binding interactions, superposition with the co-crystallized ligand, and docking score are discussed in the text.

## 4. Conclusions

In conclusion, this work aimed to design and synthesize variable structural isatin-quinazoline based compounds as potential protein kinase inhibitors. Fourteen different compounds were designed and synthesized based on a hybrid pharmacophoric design approach. The synthesis of target compounds was performed by condensation of substituted isatin derivatives **6a**–**g** with 6-aminoquinazoline to give the substituted indolyl quinazolinones **7**–**13** and with 4-(*p*-aminophenyl) quinazoline to produce the substituted indolyl phenyl quinazolinones **18**–**24** in good yields**.** Out of the fourteen compounds, compounds **9** and **20** showed potent cytotoxicity on HepG-2 and MCF-7 cells in the cytotoxicity screening. Additionally, they also exhibited promising kinase inhibitory activities, particularly against CDK-2 and VEGFR-2. Compound **9** showed moderate inhibitory activity against VEGFR-2 and EFGR kinases but strong effects against CDK-2 and CDK-4. Meanwhile, Compound **20** demonstrated potent inhibition of EFGR and VEGFR-2 along with modest CDK-2 and weak CDK-4 inhibitory action. Furthermore, DNA flow cytometric analysis demonstrated that both compounds **9** and **20** exhibited potent pro-apoptotic activity by elevation of cell population in the G_0_ phase. ELISA measurements showed upregulation of p53 and p21, suggesting their impact on cell cycle arrest at the G_1_ phase. Additionally, they also elevated the levels of FAS, Bax/Bcl-2 ratio, and caspase 3/7 in HepG-2 cells and triggered apoptosis. Molecular docking studies supported the potential binding modes and interactions of compound **9** with CDK-2 and compound **20** with VEGFR-2. The results of this study underscore the significant impact of connecting isatin with quinazoline moieties, specifically through imino or iminophenyl C6 linkage, on the observed cytotoxic activity. This structural insight suggests that the linkage of isatin and quinazoline fragments led to the development of a promising structural moiety and several novel pro-apoptotic multi-target agents as lead compounds that could be used for further development of more effective anti-cancer agents.

## Figures and Tables

**Table 1:** In vitro cytotoxicity on HepG-2 and MCF-7 cells of compounds **7**–**13** and **18**–**24** and VEGFR-2, EGFR, CDK-2, and CDK-4 enzymes inhibition of compounds **9** and **20**.

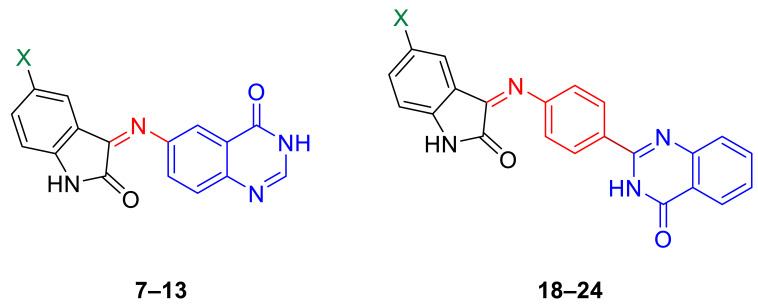							
Comp No.	X	IC50 (µM) a		IC50 (nM) a			
		HepG2 b	MCF-7 c	VEGFR-2 d	EGFR e	CDK-2 f	CDK-4 g
**7**	-H	10.75 ± 0.92	21.86 ± 1.83	ND	ND	ND	ND
**8**	-F	7.54 ± 0.53	52.83 ± 3.21	ND	ND	ND	ND
**9**	-Cl	2.53 ± 0.11	7.54 ± 0.71	56.74 ± 4.3	87.48 ± 6.71	9.39 ± 0.51	36.39 ± 4.52
**10**	-Br	28.11 ± 2.08	>100	ND	ND	ND	ND
**11**	-NO_2_	20.87 ± 1.54	72.39 ± 6.51	ND	ND	ND	ND
**12**	-CH_3_	31.89 ± 2.12	46.17 ± 2.47	ND	ND	ND	ND
**13**	-OCH_3_	18.86 ± 1.05	>100	ND	ND	ND	ND
**18**	-H	82.42 ± 7.17	>100	ND	ND	ND	ND
**19**	-F	28.37 ± 1.26	40.83 ± 3.24	ND	ND	ND	ND
**20**	-Cl	3.08 ± 0.35	5.28 ± 0.22	14.31 ± 2.70	32.65 ± 1.61	225.32 ± 12.56	244.32 ± 12.21
**21**	-Br	23.71 ± 0.74	33.56 ± 2.33	ND	ND	ND	ND
**22**	-NO_2_	35.82 ± 2.0	14.94 ± 0.66	ND	ND	ND	ND
**23**	-CH_3_	38.43 ± 2.71	15.35± 0.23	ND	ND	ND	ND
**24**	-OCH_3_	32.71 ± 2.93	18.53 ± 0.96	ND	ND	ND	ND
**Ind**	-H	6.92 ± 0.65	6.12 ± 0.35	126.42 ± 20	175.46 ± 18.33	45.60 ± 2.24	23.64 ± 2.15

^a^ values given are the mean of three experiments ± SEM, ^b^ HePG-2: human hepatocellular carcinoma, ^c^ MCF-7: breast cancer cells, ^d^ VEGFR-2: vascular endothelial growth factor receptor-2, ^e^ EFGR: epidermal growth factor receptor, ^f^ CDK-2: cyclin dependant kinase, ^g^ CDK-4: cyclin dependent kinase-4, ND: not determined.

## Data Availability

The authors declare that the data supporting the findings of this study are available within the paper and its Supplementary Information Files. Any raw data file needed in another format; they are available from the corresponding authors upon reasonable request.

## Supplementary Materials

The following supporting information can be downloaded at: https://www.mdpi.com/article/10.3390/molecules30051105/s1, Figures S1–S4: NMR spectra of compounds **9** and **20**; Tables S1 and S2: Viability percentages at eight concentrations and IC50 values for each compound on HepG-2 and MCF-7 cell lines; Table S3: VEGFR-2, EGFR, CDK-2 and CDK-4 kinase inhibition percentages of compounds **9**, **20**, and Ind; Table S4: Cell cycle distribution percentage of HePG2 carcinoma cell line upon treatment with compounds **9** and **20**; Table S5: In vitro DNA flow cytometry apoptosis analysis; Tables S6–S11: Concentrations of apoptosis-related markers in HepG-2 carcinoma cells after treatment with compounds **9**, **20**, and Ind at their IC50 values.
