# Supplementary material for: Design, Synthesis, and Cytotoxicity Evaluation of Novel Indolin-2-One Based Molecules on Hepatocellular Carcinoma HepG2 Cells as Protein Kinase Inhibitors
Source: Molecules. 2025 Feb 28;30(5):1105. doi: 10.3390/molecules30051105 (PMC11901902; doi:10.3390/molecules30051105)
Supplement: Supplementary file 1 [file molecules-30-01105-s001.zip › molecules-3335740-supplementary.pdf]

Supporting Information

Design, Synthesis, and Cytotoxicity Evaluation of Novel Indolin-2-One Based Molecules on Hepatocellular Carcinoma HepG2 Cells as Protein Kinase Inhibitors

Manal M. Kandeel [1], Mohamed Kamal AbdElhameid [1,*], Mohamed Adel [2], Muhammad Y. Al-Shorbagy [3,4] and Ahmed T. Negmeldin [1,3,*]

[1] Department of Pharmaceutical Organic Chemistry, Faculty of Pharmacy, Cairo University, Cairo 11562, Egypt

[2] Department of Pharmaceutical Organic Chemistry, Faculty of Pharmacy, Egyptian Russian University, Cairo 11829, Egypt

[3] Department of Pharmaceutical Sciences, College of Pharmacy, Gulf Medical University, Ajman 4184, United Arab Emirates
[4] Pharmacology and Toxicology Department, Faculty of Pharmacy, Cairo University, Cairo 11562, Egypt

* Correspondence: mohamed.sayed@pharma.cu.edu.eg (M.K.A.); dr.ahmedthabet@gmu.ac.ae (A.T.N.)

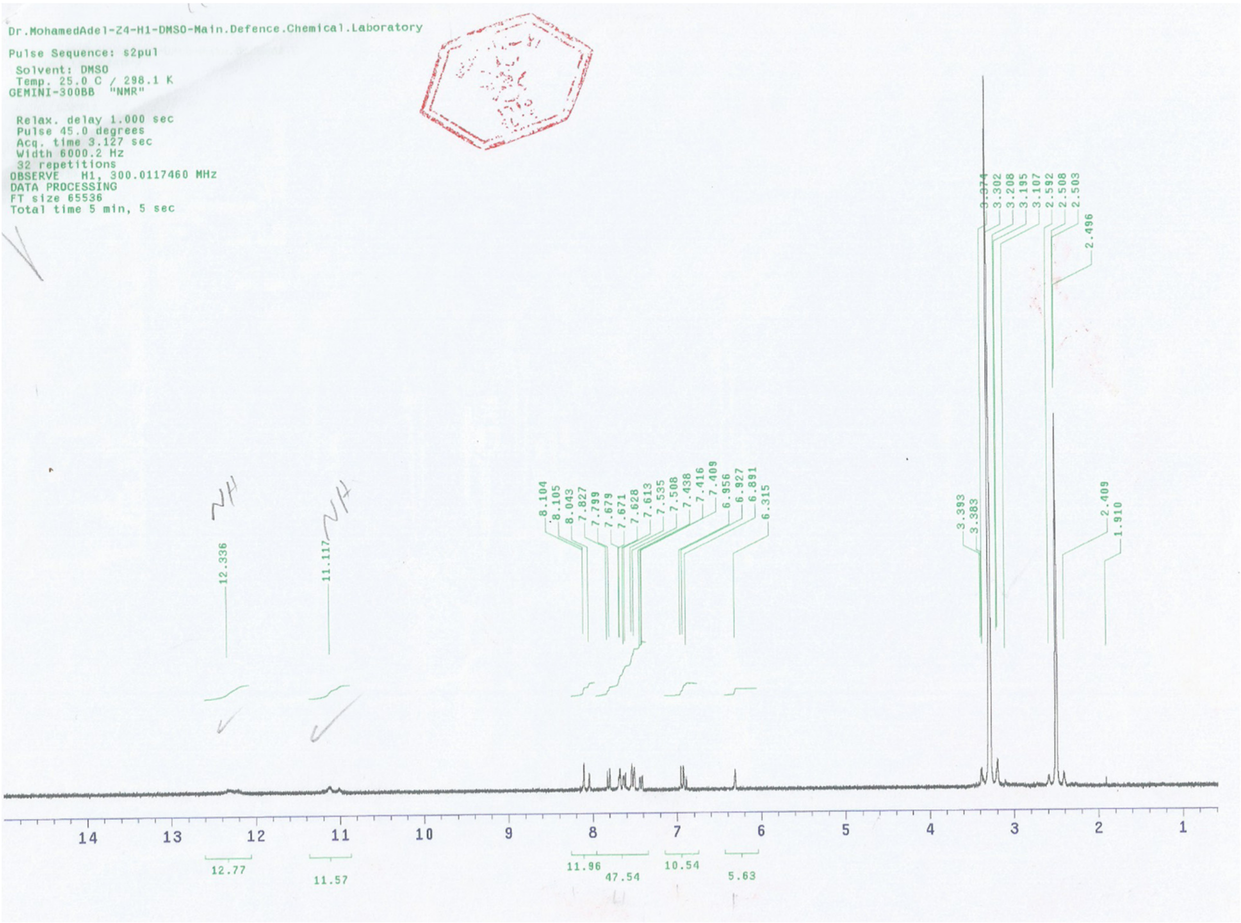

Figure S1: ^{1}HNMR spectrum of 6-(5-Chloro-2-oxo-1,2-dihydro-indol-3-ylideneamino)-3H-quinazolin-4-one (9)

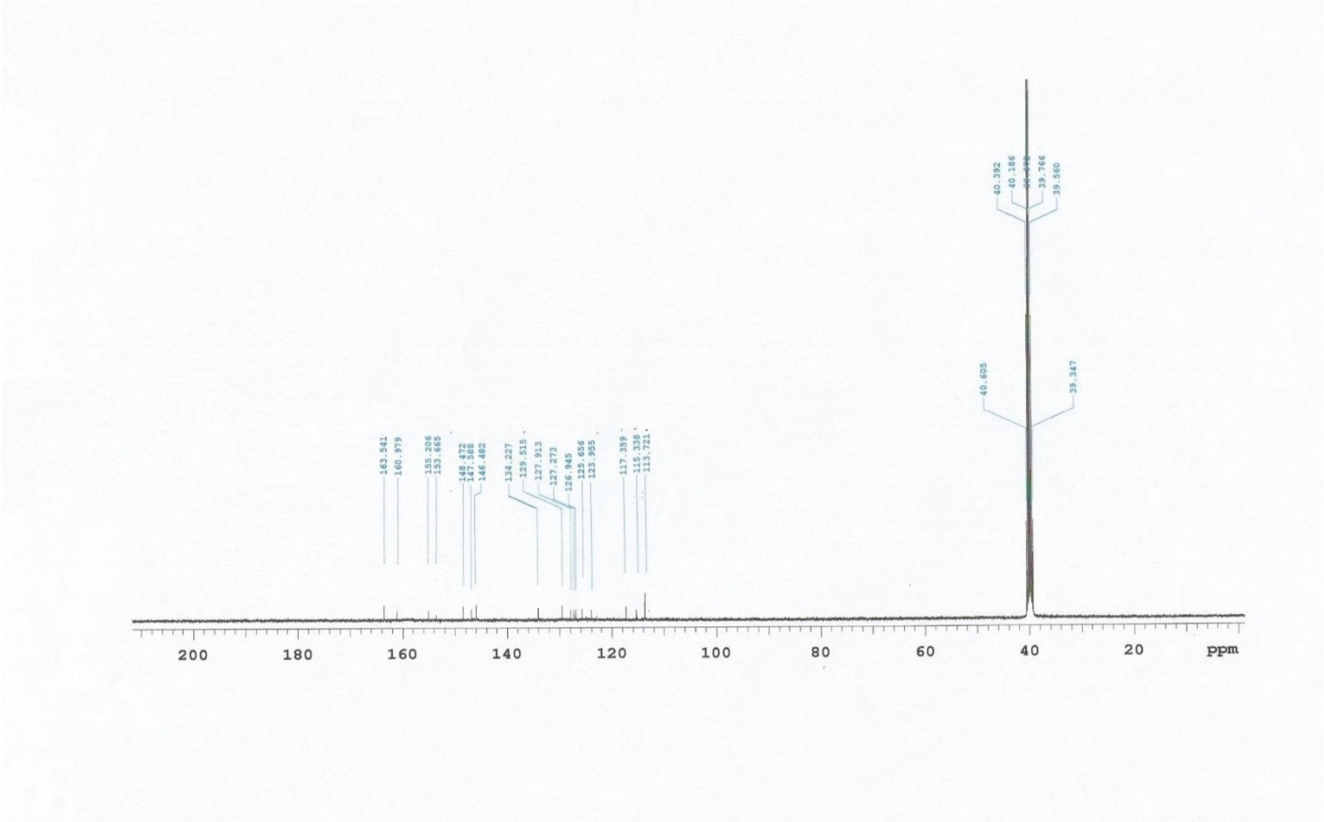

Figure S2: ^{13}CNMR spectrum of 6-(5-Chloro-2-oxo-1,2-dihydro-indol-3-ylideneamino)-3H-quinazolin-4-one (9)

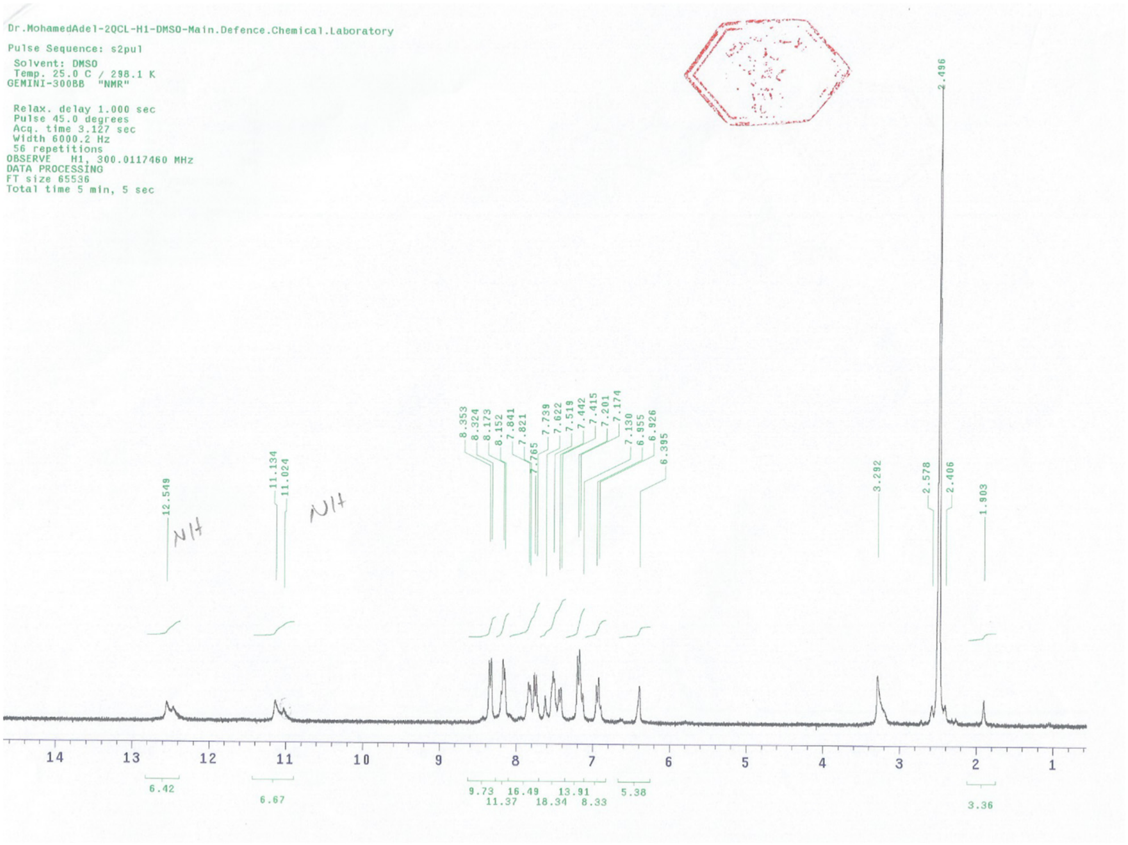

Figure S3: ^{1}HNMR spectrum of 2-[4-(5-Chloro-2-oxo-1,2-dihydro-indol-3-ylideneamino)-phenyl]-3H quinazolin-4-one (20)

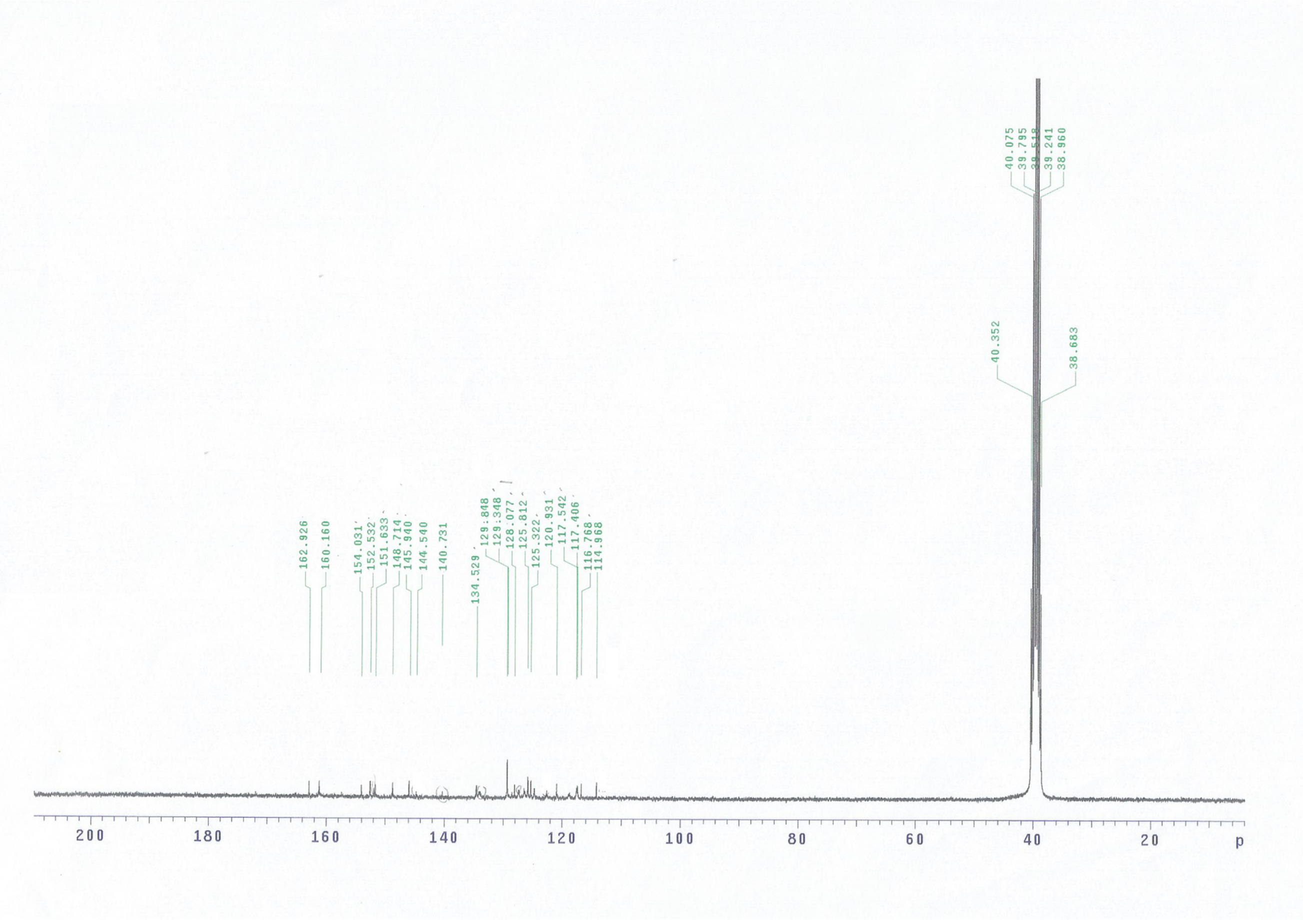

Figure S4: ^{13}CNMR spectrum of 2-[4-(5-Chloro-2-oxo-1,2-dihydro-indol -3-ylideneamino)-phenyl]-3H-quinazolin -4-one (20)

Table S1: The viability % for 8 concentration (μM) for each compound and their IC_{50} (μM) on HepG-2 cell line

	Viability % at each concentration (μM)								IC_{50}
	1.25	2.5	5	10	20	40	80	160	(μM)
**7**	83.4 ± 6.9	70.9 ± 6.0	61.9 ± 5.1	49.3 ± 2.2	35.9 ± 2.1	26.5 ± 1.9	9.4 ± 0.4	7.2 ± 0.2	10.75 ± 0.9
**8**	88 ± 7.6	71.2 ± 6.1	62.3 ± 4.3	48.2 ± 1.8	33.6 ± 0.13	0.9 ± 17.5	15.1 ± 1.4	9.3 ± 1.1	7.54 ± 0.5
**9**	61.3 ± 3.6	54.4 ± 3.1	34.8 ± 0.9	28.6 ± 2.1	23.1 ± 2.4	19.6 ± 2.0	16.3 ± 1.7	12.9 ± 1.5	2.53 ± 0.1
**10**	91.16 ± 7.0	87.9 ± 8.1	83 ± 5.2	71.4 ± 4.5	58.7 ± 4.3	35.8 ± 2.1	19.42 ± 1.2	14.19 ± 0.9	28.11 ± 2.0
**11**	99.2 ± 6.9	91.3 ± 7.9	89.43 ± 6.4	79.5 ± 5.5	67.1 ± 3.8	54.6 ± 1.9	44.1 ± 1.3	37.6 ± 1.1	20.87 ± 1.5
**12**	96.4 ± 6.9	84.9 ± 6.1	81.8 ± 5.4	69.1 ± 4.1	58.9 ± 2.2	49.7 ± 1.7	36.3 ± 0.5	33.93 ± 1.2	31.89 ± 2.1
**13**	88.2 ± 7.4	6.5 ± 86.5	75.9 ± 4.6	61.5 ± 3.2	40.9 ± 2.7	26.6 ± 1.5	7.9 ± 0.9	3.9 ± 0.3	18.86 ± 1.0
**18**	77.96 ± 7.2	75.59 ± 6.8	73.49 ± 5.8	55.89 ± 4.1	40.38 ± 2.9	24.92 ± 2.8	4.54 ± 0.23	1.95 ± 0.19	82.42 ± 7.1
**19**	68.38 ± 6.6	75.03 ± 5.3	72.32 ± 5.9	58.44 ± 3.5	46.30 ± 2.4	26.62 ± 1.7	13.61 ± 0.8	7.47 ± 0.2	28.37 ± 1.2
**20**	58.2 ± 3.3	52.1 ± 2.9	36.7 ± 1.2	29.4 ± 2.5	21.7 ± 2.0	18.4 ± 1.8	15.2 ± 1.5	11.8 ± 1.3	3.08 ± 0.3
**21**	98.30 ± 7.7	82.55 ± 6.8	77.75 ± 5.5	61.41 ± 4.6	47.04 ± 3.4	23.04 ± 1.5	9.33 ± 1.1	6.14 ± 0.9	23.71 ± 0.7
**22**	79.17 ± 7.2	73.30 ± 6.5	62.95 ± 5.9	48.46 ± 4.1	37.99 ± 3.8	26.14 ± 2.3	8.22 ± 0.5	4.17 ± 0.2	35.82 ± 2.0
**23**	81.5 ± 7.5	75.3 ± 6.8	64.8 ± 6.0	50.2 ± 4.4	39.1 ± 3.6	28.7 ± 2.6	9.5 ± 0.6	4.5 ± 0.3	38.43 ± 2.7
**24**	99.39 ± 8.2	95.51 ± 7.3	88.45 ± 7	79.59 ± 6.9	62.12 ± 5.7	49.45 ± 3.6	23.88 ± 2.4	10.43 ± 1.1	32.71 ± 2.9

Table S2: The viability % for 8 concentration (μM) for each compound and their IC_{50} (μM) on MCF-7 cell line

	Viability % at each concentration (μM)								IC50
	1.25	2.5	5	10	20	40	80	160	(μM)
**7**	101.4 ± 7.2	93.7 ± 8.2	87.6 ± 6.1	78.2 ± 5.3	66.3 ± 4.0	53.1 ± 2.0	45.2 ± 1.4	36.8 ± 1.2	21.86 ± 1.8
**8**	103.5 ± 4.9	94.22 ± 4	87.92 ± 3.9	75.71 ± 3.2	57.14 ± 2.4	43.91 ± 2	29.70 ± 1.4	11.54 ± 0.7	52.83 ± 3.2
**9**	90.5 ± 7.8	72.8 ± 6.3	64.1 ± 4.5	49.0 ± 2.0	34.4 ± 0.2	18.2 ± 1.0	14.8 ± 1.3	8.7 ± 1.2	7.54 ± 0.7
**10**	98.3 ± 7.4	99.1 ± 6.9	87.2 ± 5.7	76.5 ± 4.0	65.2 ± 2.8	55.4 ± 2.6	41.2 ± 2.8	25.4 ± 2.6	112 ± 11.2
**11**	116.02 ± 6.4	97.47 ± 7	90.06 ± 6.4	86.04 ± 5.5	63.85 ± 3.8	39.15 ± 2.9	25.55 ± 1.3	11.19 ± 0.4	72.39 ± 6.5
**12**	103.75 ± 5.4	91.36 ± 4.1	79.97 ± 4.2	69.21 ± 2.7	58.49 ± 1.5	30.15 ± 1.2	21.49 ± 1.5	9.15 ± 1.2	46.17 ± 2.4
**13**	108.81 ± 7.3	107.57 ± 7.4	99.62 ± 6.5	87.62 ± 4.6	71.93 ± 3.2	67.64 ± 2.7	60.80 ± 1.5	56.82 ± 0.9	123 ± 13.2
**18**	109.07 ± 6.2	92 ± 6.8	94.175 ± 5.8	80.8 ± 4.9	84.85 ± 2.9	83.75 ± 2.8	74.30 ± 1.2	67.88 ± 0.8	143 ± 16.2
**19**	109.15 ± 6.6	99.33 ± 5.3	93.92 ± 4.9	81.15 ± 3.9	59.04 ± 2.4	36.44 ± 1.7	16.43 ± 1.8	11.96 ± 1.2	40.83 ± 3.2
**20**	92.36 ± 5.7	84.32 ± 4.8	76.73 ± 4.2	67.88 ± 3.4	47.07 ± 3	24.56 ± 1.9	7.77 ± 0.9	4.10 ± 0.1	5.28 ± 0.2
**21**	102.78 ± 7.7	99.57 ± 6.8	91.59 ± 5.5	78.22 ± 4.6	66.32 ± 3.4	33.16 ± 2.5	15.98 ± 1.1	10.34 ± 0.9	33.56 ± 2.3
**22**	109.15 ± 8	99.57 ± 7.4	95.32 ± 5.9	79.07 ± 5.1	63.58 ± 3.8	49.92 ± 3.3	23.47 ± 2.5	12.95 ± 1.3	14.94 ± 0.6
**23**	101.34 ± 7.9	91.49 ± 6.5	78.82 ± 6.4	65.75 ± 5.6	40.86 ± 4.6	26.85 ± 3	12.04 ± 1.3	11.96 ± 1.6	15.35 ± 0.2
**24**	103.75 ± 8.4	97.94 ± 7.7	90.79 ± 7	80.05 ± 6.9	61.32 ± 4.7	29.60 ± 2.6	11.14 ± 1.4	5.92 ± 0.5	18.53 ± 0.9

Table S3 : VEGFR-2, EGFR ,CDK-2 and CDK-4 kinase inhibition % of synthetic molecules 9, 20, and Ind

Code	conc.nM	log conc	%inh	T2	T1	ΔT	RFU2	RFU1	ΔRFU	slope	K.Activity	EC
s1	1000	4	67.17398	30	0	30	0.539	0	0.539	0.054733	39.39123	120
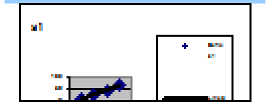	100	3	45.24936	30	0	30	0.899	0	0.899	0.054733	65.70077	120
	10	2	21.92401	30	0	30	1.282	0	1.282	0.054733	93.69119	120
	1	1	10.8399	30	0	30	1.464	0	1.464	0.054733	106.9921	120
EC			0	30	0	30	1.642	0	1.642	0.054733	120	120
Code<br>S2	conc.nM	log conc	%inh	T2	T1	ΔT	RFU2	RFU1	ΔRFU	slope	K.Activity	EC
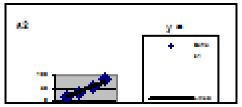	1000	4	81.05957	30	0	30	0.311	0	0.311	0.054733	22.72852	120
	100	3	53.8365	30	0	30	0.758	0	0.758	0.054733	55.3962	120
	10	2	34.28705	30	0	30	1.079	0	1.079	0.054733	78.85554	120
	1	1	13.03236	30	0	30	1.428	0	1.428	0.054733	104.3612	120
EC			0	30	0	30	1.642	0	1.642	0.054733	120	120
Code	conc.nM	log conc	%inh	T2	T1	ΔT	RFU2	RFU1	ΔRFU	slope	K.Activity	EC
s3	1000	4	73.02054	30	0	30	0.443	0	0.443	0.054733	32.37535	120
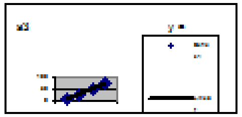	100	3	50.1215	30	0	30	0.819	0	0.819	0.054733	59.8542	120
	10	2	27.22246	30	0	30	1.195	0	1.195	0.054733	87.33305	120
	1	1	9.804566	30	0	30	1.481	0	1.481	0.054733	108.2345	120
			0	30	0	30	1.642	0	1.642	0.054733	120	120
Code<br>S1	conc.nM	log conc	%inh	T2	T1	ΔT	RFU2	RFU1	ΔRFU	slope	K.Activity	EC
	1000	4	62.05823	30	0	30	0.623	0	0.623	0.054733	45.53012	120
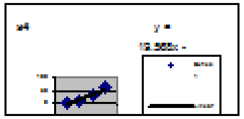												

s4												
	100	3	30.14574	30	0	30	1.147	0	1.147	0.054733	83.82511	120
	10	2	12.42334	30	0	30	1.438	0	1.438	0.054733	105.092	120
	1	1	2.739968	30	0	30	1.597	0	1.597	0.054733	116.712	120
EC			0	30	0	30	1.642	0	1.642	0.054733	120	120

Code<br>S2	conc.nM	log conc	%inh	T2	T1	ΔT	RFU2	RFU1	ΔRFU	slope	K.Activity	EC
	1000	4	84.22646	30	0	30	0.259	0	0.259	0.054733	18.92825	120
	100	3	56.27257	30	0	30	0.718	0	0.718	0.054733	52.47291	120
	10	2	36.41861	30	0	30	1.044	0	1.044	0.054733	76.29766	120
	1	1	18.57441	30	0	30	1.337	0	1.337	0.054733	97.7107	120
EC			0	30	0	30	1.642	0	1.642	0.054733	120	120

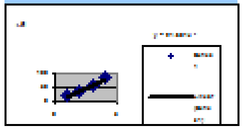

Code<br>S3	conc.nM	log conc	%inh	T2	T1	ΔT	RFU2	RFU1	ΔRFU	slope	K.Activity	EC
	1000	4	80.02424	30	0	30	0.328	0	0.328	0.054733	23.97091	120
	100	3	57.79511	30	0	30	0.693	0	0.693	0.054733	50.64586	120
	10	2	31.48558	30	0	30	1.125	0	1.125	0.054733	82.21731	120
	1	1	12.17973	30	0	30	1.442	0	1.442	0.054733	105.3843	120
			0	30	0	30	1.642	0	1.642	0.054733	120	120

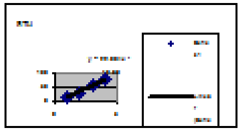

Code	conc.nM	log conc	%inh	T2	T1	ΔT	RFU2	RFU1	ΔRFU	slope	K.Activity	EC
s1	1000	4	84.44704	30	0	30	39875	0	39875	8546.067	18.66356	120
	100	3	63.8633	30	0	30	92648	0	92648	8546.067	43.36404	120
	10	2	40.37296	30	0	30	152873	0	152873	8546.067	71.55245	120
	1	1	11.55698	30	0	30	226752	0	226752	8546.067	106.1316	120

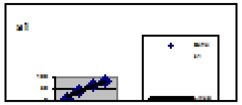

EC			0	30	0	30	256382	0	256382	8546.067	120	120
Code												
S2	conc.nM	log conc	%inh	T2	T1	ΔT	RFU2	RFU1	ΔRFU	slope	K.Activity	EC
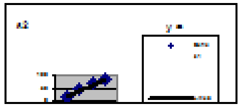	1000	4	81.84896	30	0	30	46536	0	46536	8546.067	21.78125	120
	100	3	65.84628	30	0	30	87564	0	87564	8546.067	40.98447	120
	10	2	44.3514	30	0	30	142673	0	142673	8546.067	66.77832	120
	1	1	14.28221	30	0	30	219765	0	219765	8546.067	102.8614	120
EC			0	30	0	30	256382	0	256382	8546.067	120	120
Code												
S3	conc.nM	log conc	%inh	T2	T1	ΔT	RFU2	RFU1	ΔRFU	slope	K.Activity	EC
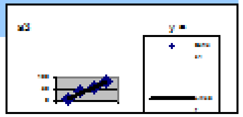	1000	4	85.57153	30	0	30	36992	0	36992	8546.067	17.31416	120
	100	3	59.57517	30	0	30	103642	0	103642	8546.067	48.5098	120
	10	2	38.39817	30	0	30	157936	0	157936	8546.067	73.92219	120
	1	1	5.558506	30	0	30	242131	0	242131	8546.067	113.3298	120
EC			0	30	0	30	256382	0	256382	8546.067	120	120
Code												
S1	conc.nM	log conc	%inh	T2	T1	ΔT	RFU2	RFU1	ΔRFU	slope	K.Activity	EC
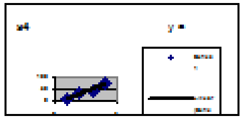	1000	4	78.39396	30	0	30	55394	0	55394	8546.067	25.92725	120
	100	3	45.61397	30	0	30	139436	0	139436	8546.067	65.26324	120
	10	2	31.14845	30	0	30	176523	0	176523	8546.067	82.62187	120
	1	1	9.22725	30	0	30	232725	0	232725	8546.067	108.9273	120
EC			0	30	0	30	256382	0	256382	8546.067	120	120
Code												
S2	conc.nM	log conc	%inh	T2	T1	ΔT	RFU2	RFU1	ΔRFU	slope	K.Activity	EC
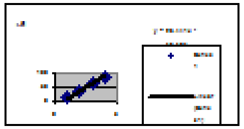	1000	4	88.36853	30	0	30	29821	0	29821	8546.067	13.95777	120

	100	3	66.32291	30	0	30	86342	0	86342	8546.067	40.41251	120
	10	2	34.339	30	0	30	168343	0	168343	8546.067	78.7932	120
	1	1	10.8939	30	0	30	228452	0	228452	8546.067	106.9273	120
EC			0	30	0	30	256382	0	256382	8546.067	120	120
Code												
S3	conc.nM	log conc	%inh	T2	T1	ΔT	RFU2	RFU1	ΔRFU	slope	K.Activity	EC
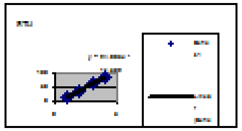	1000	4	82.96838	30	0	30	43666	0	43666	8546.067	20.43794	120
	100	3	63.90425	30	0	30	92543	0	92543	8546.067	43.3149	120
	10	2	35.02976	30	0	30	166572	0	166572	8546.067	77.96428	120
	1	1	12.57772	30	0	30	224135	0	224135	8546.067	104.9067	120
EC			0	30	0	30	256382	0	256382	8546.067	120	120

Table S4: Cell cycle distribution % of HePG2 carcinoma cell line of compounds 9 and 20.

Cell Cycle Distribution (%)

Comp No	(IC_{50}µM)	sub-G0/G1	%G0-G1	%S	%G2-M
S1	**2.50**	13.01	45.22	32.03	20.75
S2	**3.08**	10.72	67.03	16.27	2.86
**Cont.HepG-2**		2.32	45.43	37.16	15.06

Table S5: *In* vitro DNA- flow cytometry apoptosis analysis

		Apoptosis			Necrosis
		Total	Early	Late	
S1	s1/HepG-2	8.77	5.05	3.72	2.69
S2	s2/ HepG-2	11.66	4.91	6.75	1.91
	cont. HepG-2	1.13	0.81	0.32	0.29

Table S6 : Concentration(pg/ml) of p53 in HepG-2 cell carcinomia after treatment with synthetic molecules 9, 20 and Ind at their IC_{50}

Sample			Results
			HepG-2
S	Code	Mw	P53<br>pg/ml± S.E.M
1	s1/ HepG-2	324	325.11±29.55
2	s2/ HepG-2	400	127.43±9.43
3	cont. HepG-2	---	48.29±3.87

Table S7 : Concentration(pg/ml) of p21 in HepG-2 cell carcinomia after treatment with synthetic molecules 9, 20 and Ind at their IC_{50}

Sample			Results
			HepG-2
S	Code	Mw	P21<br>pg/ml± S.E.M

1	s1/ HepG-2	324	91.44±8.65
2	s2/ HepG-2	400	105.67±9.88
3	cont. HepG-2	---	23.33±3.05

Table S8 : Concentration(pg/ml) of Fas L in HepG-2 cell carcinomia after treatment with synthetic molecules 9, 20 and INd at their IC_{50}

Sample			Results
			HepG-2
S	Code	Mw	FasL<br>pg/ml± S.E.M
1	S1/ HepG-2	324	86.11±8.05
2	S2/ HepG-2	400	62.06±4.62
3	cont. HepG-2	---	15.33±1.71

Table S9: Concentration(pg/ml) of Bax in HepG-2 cell carcinomia after treatment with synthetic molecules 9, 20 and Ind at their IC_{50}

Sample			Results
			HepG-2
S	Code	Mw	BAX<br>pg/ml± S.E.M
1	s1/ HepG-2	324	88.12±5.27
2	s2/ HepG-2	400	108.32±9.82
3	cont. HepG-2	---	18.93±11.71

Table S10 : Concentration(ng/lml) of Bcl-2 in HepG-2 cell carcinomia after treatment with synthetic molecules 9, 20 and Ind at their IC_{50}

Sample			Results
			HepG-2
S	Code	Mw	BCl-2<br>pg/ml± S.E.M
1	s1/ HepG-2	324	90.15±8.27
2	s2/ HepG-2	400	160.32±10.82
3	cont. HepG-2	---	360.90±31.71

Table S11 : Concentration(pg/ml) of caspases 3/7 in HepG-2 cell carcinomia after treatment with synthetic molecules 9, 20 and Ind at their IC_{50}

Sample			Results
			HepG-2
S	Code	Mw	Caspase3/7 pg/ml± S.E.M
1	s1/ HepG-2	324	62.13±5.27
2	s2/ HepG-2	400	37.32±4.82
3	cont. HepG-2	---	11.90±1.71

Detailed Results:

STANDARDS	CONC.pg/ml
St.1	1000
St.2	500
St.3	250
St.4	125
St.5	62.5
St.6	31.2
St.7	15.6

Plate map

	1	2	3	4	5	6	7	8	9	10	11	12
A	St.1	s1	s3	Stu	--	--	--	--	--	--	--	--
B	St.2	s1	s4	Stu	--	--	--	--	--	--	--	--
C	St.3	s1	s4	C	--	--	--	--	--	--	--	--
D	St.4	s2	s4	C	--	--	--	--	--	--	--	--
E	St.5	s2	s5	C	--	--	--	--	--	--	--	--
F	St.6	s2	s5	B	--	--	--	--	--	--	--	--
G	St.7	s3	s5	--	--	--	--	--	--	--	--	--
H	B	s3	Stu	--	--	--	--	--	--	--	--	--

Samples OD results

	1	2	3	4	5	6	7	8	9	10	11	12
A	2.251	0.392	0.387	0.346	0	0	0	0	0	0	0	0
B	1.154	0.402	0.434	0.361	0	0	0	0	0	0	0	0
C	0.709	0.387	0.429	0.767	0	0	0	0	0	0	0	0
D	0.348	0.316	0.451	0.783	0	0	0	0	0	0	0	0

E	0.233	0.302	0.292	0.759	0	0	0	0	0	0	0	0
F	0.134	0.288	0.296	0.003	0	0	0	0	0	0	0	0
G	0.086	0.413	0.309	0	0	0	0	0	0	0	0	0
H	0.015	0.394	0.357	0	0	0	0	0	0	0	0	0

**Calibrator**	**Wells**	**Conc.**	**Raw (Corrected)**	**Backfit**	**Recovery %**
Standard1	A1	1000	2.24	1005	100.5
Standard2	B1	500	1.15	478.5	95.7
Standard3	C1	250	0.7	274.6	109.8
Standard4	D1	125	0.339	116.6	93.32
Standard5	E1	62.5	0.224	68.82	110.1
Standard6	F1	31.25	0.125	29.46	94.26
Standard7	G1	15.63	0.077	11.5	73.6

**Sample**	**Wells**	**Raw**	**Background Corrected**	**Conc.**	**Conc. (Average)**	**%CV**	**SD**	**SEM**
Control	C4<br>D4<br>E4	0.767<br>0.783<br>0.759	0.761	300.7<br>307.9<br>297.1	301.9	1.83	5.52	3.19
s1/HepG-2	A2<br>B2<br>C2	0.392<br>0.402<br>0.387	0.385	135.4<br>139.6<br>133.2	136.1	2.4	3.27	1.89
s2/HepG-2	D2<br>E2<br>F2	0.316<br>0.302<br>0.288	0.293	103.2<br>97.31<br>91.48	97.32	6.01	5.85	3.38
s3/HepG-2	A3<br>G2<br>H2	0.387<br>0.413<br>0.394	0.389	133.2<br>144.3<br>136.2	137.9	4.18	5.76	3.33
S1/HepG-2	B3<br>C3<br>D3	0.434<br>0.429<br>0.451	0.429	153.4<br>151.2<br>160.7	155.1	3.21	4.98	2.87
S2/HepG-2	E3<br>F3<br>G3	0.292<br>0.296<br>0.309	0.29	93.14<br>94.81<br>100.2	96.06	3.86	3.71	2.14
S3/ HepG-2	A4<br>B4<br>H3	0.346<br>0.361<br>0.357	0.346	115.8<br>122.2<br>120.5	119.5	2.75	3.29	1.9
Blank	F4<br>H1	0.003<br>0.015	0	< Curve<br>< Curve	-	-	-	-

Results:

ST.	CONC.%
St.1	1000
St.2	500
St.3	250
St.4	125
St.5	62.5
St.6	31.2
St.7	16.1

Detailed Results:

	1	2	3	4	5	6	7	8	9	10	11	12
A	St.1	s1	s3	Stu	0	0	0	0	0	0	0	0
B	St.2	s1	s4	Stu	0	0	0	0	0	0	0	0
C	St.3	s1	s4	C	0	0	0	0	0	0	0	0
D	St.4	s2	s4	C	0	0	0	0	0	0	0	0
E	St.5	s2	s5	C	0	0	0	0	0	0	0	0
F	St.6	s2	s5	B	0	0	0	0	0	0	0	0
G	St.7	s3	s5	0	0	0	0	0	0	0	0	0
H	B	s3	stu	0	0	0	0	0	0	0	0	0

Samples OD results

	1	2	3	4	5	6	7	8	9	10	11	12
A	2.294	0.472	0.348	0.315	0	0	0	0	0	0	0	0
B	1.338	0.449	0.513	0.337	0	0	0	0	0	0	0	0
C	0.814	0.463	0.541	0.695	0	0	0	0	0	0	0	0
D	0.663	0.295	0.522	0.709	0	0	0	0	0	0	0	0
E	0.482	0.332	0.267	0.728	0	0	0	0	0	0	0	0
F	0.367	0.313	0.288	0.012	0	0	0	0	0	0	0	0
G	0.159	0.372	0.292	0	0	0	0	0	0	0	0	0
H	0.018	0.365	0.331	0	0	0	0	0	0	0	0	0

Calibrator	Wells	Conc.	Raw (Corrected)	Backfit	Recovery %
Standard1	A1	1000	2.28	1013	101.3
Standard2	B1	500	1.32	477.7	95.55
Standard3	C1	250	0.799	223.5	89.39
Standard4	D1	125	0.648	158	126.4
Standard5	E1	62.5	0.467	85.95	137.5
Standard6	F1	31.25	0.352	45.28	144.9
Standard7	G1	15.63	0.144	< Curve	-

.

Sample	Wells	Raw	Background Corrected	Conc.	Conc. (Average)	%CV	SD	SEM
Control	C4<br>D4<br>E4	0.695<br>0.709<br>0.728	0.696	171.5<br>177.5<br>185.6	178.2	3.99	7.11	4.1
s1/<br>HepG-2	A2<br>B2<br>C2	0.472<br>0.449<br>0.463	0.446	82.23<br>73.79<br>78.9	78.31	5.43	4.25	2.45
s2/HepG-2	D2<br>E2<br>F2	0.295<br>0.332<br>0.313	0.298	22.8<br>33.99<br>28.14	28.31	19.8	5.6	3.23
s3/H<br>epG-2	A3<br>G2<br>H2	0.348<br>0.372<br>0.365	0.347	39.08<br>46.94<br>44.62	43.54	9.28	4.04	2.33
S1/HepG-2	B3<br>C3<br>D3	0.513<br>0.541<br>0.522	0.51	97.67<br>108.5<br>101.1	102.4	5.4	5.53	3.19
S2/HepG-2	E3<br>F3<br>G3	0.267<br>0.288<br>0.292	0.267	14.96<br>20.78<br>21.93	19.23	19.4	3.74	2.16
S3/HepG-2	A4<br>B4<br>H3	0.315<br>0.337<br>0.331	0.313	28.75<br>35.57<br>33.68	32.66	10.8	3.52	2.03
Blank	F4<br>H1	0.012<br>0.018	0	< Curve<br>< Curve	-	-	-	-
